# Investigating the environmental drivers of deep‐seafloor biodiversity: A case study of peracarid crustacean assemblages in the Northwest Atlantic Ocean

**DOI:** 10.1002/ece3.5852

**Published:** 2019-11-27

**Authors:** Oliver S. Ashford, Andrew J. Kenny, Christopher R. S. Barrio Froján, Tammy Horton, Alex D. Rogers

**Affiliations:** ^1^ Department of Zoology University of Oxford Oxford UK; ^2^ Centre for the Environment, Fisheries and Aquaculture Science (Cefas) Lowestoft UK; ^3^ Seascape Consultants Ltd Romsey UK; ^4^ National Oceanography Centre University of Southampton Waterfront Campus Southampton UK; ^5^ Present address: Scripps Institution of Oceanography La Jolla CA USA

**Keywords:** benthic ecology, current speed, deep sea, food availability, functional diversity, habitat heterogeneity, macrofauna, Peracarida, phylogenetic diversity, sediment characteristics, temperature, temporal variability, trawling

## Abstract

The deep‐sea benthos covers over 90% of seafloor area and hosts a great diversity of species which contribute toward essential ecosystem services. Evidence suggests that deep‐seafloor assemblages are structured predominantly by their physical environment, yet knowledge of assemblage/environment relationships is limited. Here, we utilized a very large dataset of Northwest Atlantic Ocean continental slope peracarid crustacean assemblages as a case study to investigate the environmental drivers of deep‐seafloor macrofaunal biodiversity. We investigated biodiversity from a phylogenetic, functional, and taxonomic perspective, and found that a wide variety of environmental drivers, including food availability, physical disturbance (bottom trawling), current speed, sediment characteristics, topographic heterogeneity, and temperature (in order of relative importance), significantly influenced peracarid biodiversity. We also found deep‐water peracarid assemblages to vary seasonally and interannually. Contrary to prevailing theory on the drivers of deep‐seafloor diversity, we found high topographic heterogeneity (at the hundreds to thousands of meter scale) to negatively influence assemblage diversity, while broadscale sediment characteristics (i.e., percent sand content) were found to influence assemblages more than sediment particle‐size diversity. However, our results support other paradigms of deep‐seafloor biodiversity, including that assemblages may vary inter‐ and intra‐annually, and how assemblages respond to changes in current speed. We found that bottom trawling negatively affects the evenness and diversity of deep‐sea soft‐sediment peracarid assemblages, but that predicted changes in ocean temperature as a result of climate change may not strongly influence continental slope biodiversity over human timescales, although it may alter deep‐sea community biomass. Finally, we emphasize the value of analyzing multiple metrics of biodiversity and call for researchers to consider an expanded definition of biodiversity in future investigations of deep‐ocean life.

## Graphical abstract

We utilize a very large dataset of Northwest Atlantic Ocean peracarid crustacean assemblages as a case study to investigate the environmental drivers of deep‐seafloor macrofaunal biodiversity. We investigate biodiversity from a phylogenetic, functional, and taxonomic perspective, and find food availability, physical disturbance (bottom trawling), current speed, sediment characteristics, topographic heterogeneity, and temperature to significantly influence peracarid biodiversity (in order of relative importance). Our results both challenge and lend support to paradigms of deep‐seafloor biodiversity. We emphasize the value of analyzing multiple metrics of biodiversity and call for researchers to consider an expanded definition of biodiversity in future investigations of deep‐ocean life.

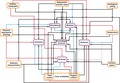

## 1 INTRODUCTION

The ocean represents Earth's largest biome, covering an area of over 440 million km^2^ and with a volume of more than 1.34 billion km^3^ (Costello, Cheung, & Hauwere, 2010; Danovaro, Snelgrove, & Tyler, 2014). Of this total, the deep ocean represents 99% of water volume and covers more than 90% of seafloor area (Costello et al., 2010). This huge biome houses significant reservoirs of biodiversity and contributes substantially to a total marine species richness of between 0.5 and 2.2 million species (Appeltans et al., 2012; Costello, Wilson, & Houlding, 2012; Mora, Tittensor, Adl, Simpson, & Worm, 2011). It has been demonstrated that deep‐sea macrofaunal assemblages are shaped predominantly by variation in their physical environment, as opposed to by interspecific competition (Ashford et al., 2018), and so knowledge of the relationships between macrofaunal assemblages and aspects of their physical environment is highly relevant to the understanding of deep‐sea diversity, and the provision of ecosystem services by deep‐ocean communities.

**Figure 1:**
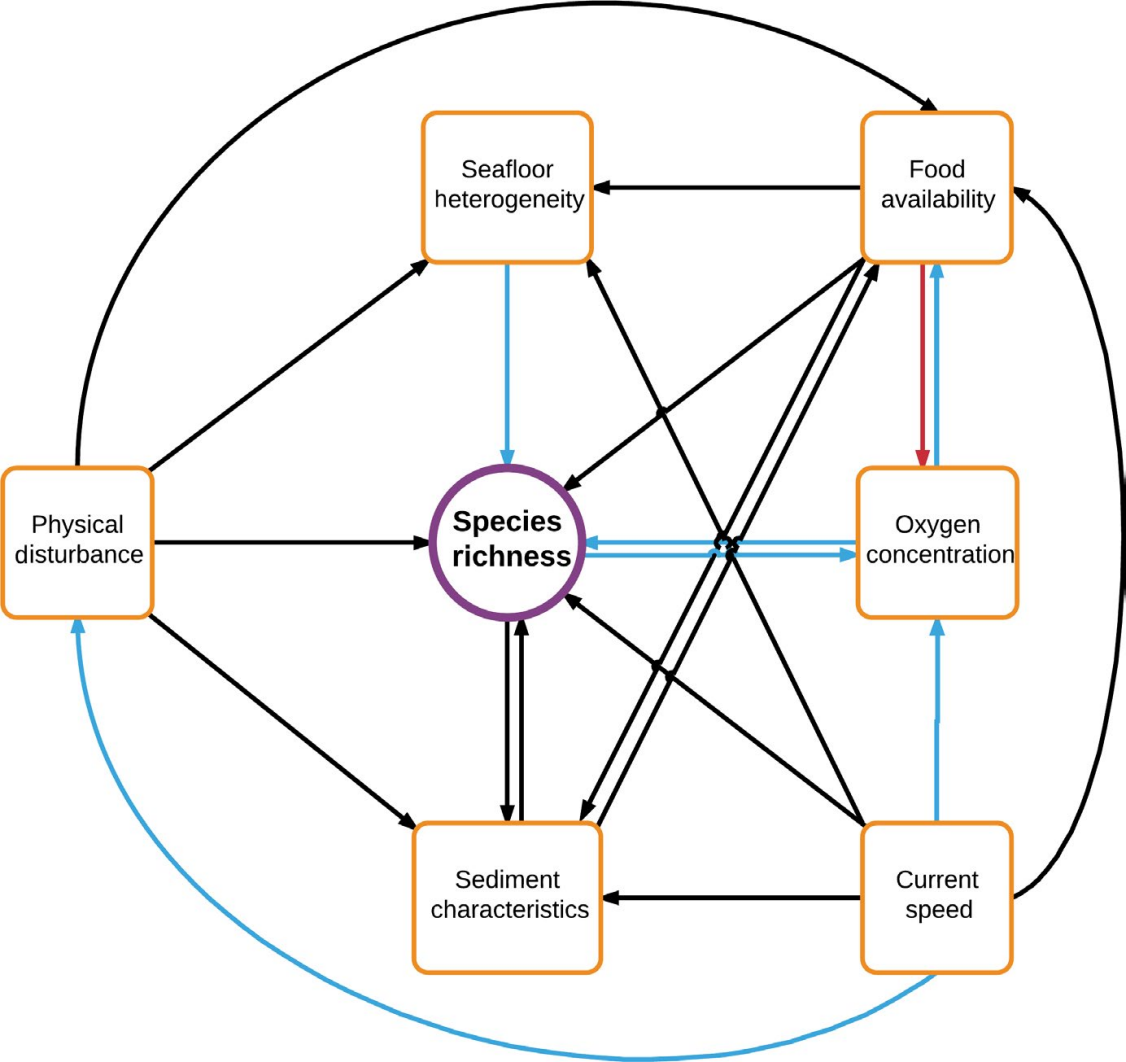
Conceptual model, specified by Levin et al. (2001), indicating the direct and indirect links between various environmental parameters (orange) and the taxonomic richness of deep‐sea communities (purple). Color of arrow represents form of relationship: blue = positive, red = negative, black = complex (e.g., “U”‐shaped or uni/multimodal)

Levin et al. (2001) presented a conceptual model (Figure 1) summarizing knowledge and hypotheses concerning the relationship between species richness in bathyal and abyssal sediments and a selection of environmental variables, including food supply, habitat heterogeneity, sediment characteristics, disturbance, oxygen concentration, and current speed. Evidence for the form of this model was generally limited to small‐scale observational and theoretical studies considering a small number of environmental variables.

Here, we utilize the study of Levin et al. (2001), among others, to help define hypotheses of relationships between a broad range of environmental variables (introduced below) and a variety of metrics of peracarid biodiversity. Specifically, we investigate twelve aspects of the attributes of benthic peracarid assemblages sampled from continental slope depths in the Northwest Atlantic Ocean, including phylogenetic and functional facets of biodiversity, assemblage density and biomass, and taxonomic metrics of diversity and assemblage structure (Table 1). We quantify functional trait and phylogenetic diversity alongside more typical diversity metrics in an attempt to capture an expanded concept of “biodiversity,” which we believe is important in order to gain a more complete understanding of the environmental controls on deep‐seafloor biodiversity.

**Table 1:** Details of biodiversity metrics analyzed

Peracarid assemblage metric	Details
Abundance	Total number of peracarid individuals per sample
Biomass	Total biomass of peracarids per sample
Rao's Quadratic Entropy	Taxonomic diversity measure considering both the number of peracarid genera per box core and their relative abundances. Simplifies to Simpson's diversity (1–*D*) for the case of taxonomic diversity. Represents the probability that two individuals taken from a sample will belong to different genera
Shannon Diversity	Taxonomic diversity measure considering both the number of peracarid genera per box core and their relative abundances. Represents a quantification of the uncertainty associated with predicting the taxonomic identity of an individual taken at random from a sample
Taxonomic Richness	Total number of peracarid genera per sample
Phylogenetic Richness (PR)	“Phylogenetic Species Variability” multiplied by the richness of peracarid genera per sample. See Helmus et al. (2007)
Functional Richness (FR)	“Functional Species Variability” multiplied by the richness of peracarid families per sample
Pielou's Index	Taxonomic evenness measure which represents the Shannon diversity value of an assemblage divided by the maximum possible value of Shannon diversity for that assemblage if all species were equally abundant
Phylogenetic Evenness (PE)	“Phylogenetic Species Variability” modified to incorporate the relative abundances of peracarid taxa per sample. See Helmus et al. (2007)
Functional Evenness (FE)	“Functional Species Variability” modified to incorporate the relative abundances of peracarid taxa per sample
Assemblage structure	Taxonomic identity of peracarid taxa present in a sample and their relative abundances

### 1.1 Topography

Topographic variation over multiple spatial scales is thought to be important in promoting high diversity in deep‐sea benthic communities (Levin, Sibuet, Gooday, Smith, & Vanreusel, 2010), and Levin et al. (2001) specified a positive linear relationship between the two variables (Figure 1). Efforts to quantify the detailed form of this association in the deep sea have been relatively limited (Levin et al., 2010), with only a few studies considering it in detail (Durden, Bett, Jones, Huvenne, & Ruhl, 2015; Morris et al., 2016; Simon‐Lledó et al., 2019; Stefanoudis, Bett, & Gooday, 2016; Vanreusel et al., 2010).

At the scale of hundreds to thousands of meters, deep‐seafloor topographic heterogeneity is greatest mid‐slope along continental margins, where canyons, cliffs, banks, ridges, mounds, gullies, and broad slopes can all be found within a relatively limited area (Levin & Dayton, 2009; Levin & Sibuet, 2012; Levin et al., 2010). These geological features are associated with distinct environmental conditions, such as sediment characteristics, food availability, current speed, temperature, and natural and anthropogenic disturbance frequency (Levin & Sibuet, 2012). As a result, different geological environments (e.g., canyons vs. banks and hills) have been proposed to support contrasting faunal assemblages (Buhl‐Mortensen et al., 2010; Duffy, Lundsten, Kuhnz, & Paull, 2014; Durden et al., 2015; Morris et al., 2016; Simon‐Lledó et al., 2019).

Studies considering the relationship between large‐scale topographic features, such as canyons, banks, hills, ridges, mounds, and gullies, and benthic faunal attributes at bathyal depths have largely focused on canyon systems, and report raised faunal assemblage density and biomass, and altered diversity patterns and assemblage structure in canyons relative to surrounding slope habitats (Bernardino, Gama, Mazzuco, Omena, & Lavrado, 2019; Danovaro, Bianchelli, Gambi, Mea, & Zeppilli, 2009; De Leo, Smith, Rowden, Bowden, & Clark, 2010; Vetter & Dayton, 1998). These studies link the biological signature of canyon environments to their tendency to entrain organic detritus from shallower waters (Levin & Sibuet, 2012). In abyssal environments, recent work has demonstrated increased taxon richness and faunal biomass on hills relative to plains or troughs for both meiofauna and megafauna (Durden et al., 2015; Morris et al., 2016; Simon‐Lledó et al., 2019; Stefanoudis et al., 2016), these studies ascribing their observations to elevated food availability on hills relative to plains as a result of topographically enhanced current speeds. We hypothesize that peracarid diversity will be positively correlated with habitat heterogeneity and that peracarid diversity, density, and biomass will be enhanced on ridges and in canyons relative to surrounding slope habitats (Table 2).

**Table 2:** Summary of environmental facets investigated, variables utilized as proxies for these facets, hypotheses relating to the influence of these facets on deep‐sea peracarid assemblages, and results of this investigation

Environmental facet	Variables used as proxies	Hypotheses	Summary of results
Topography	Seafloor rugosity/roughness; geological environment; Bathymetric Position Index	Positive relationship between variability in seafloor topography and peracarid biodiversity metrics; peracarid abundance, biomass, biodiversity metrics, and assemblage structure will vary with geological environment; peracarid abundance, biomass, and biodiversity metrics will be enhanced on ridges and in canyons relative to surrounding slope habitats	Negative relationship between peracarid biodiversity metrics and variability in seafloor topography; peracarid assemblage structure varies with geological environment and seafloor topographic variation; peracarid abundance depressed on steep slopes; peracarid biomass and biodiversity metrics enhanced on ridges relative to valleys
Sediment characteristics	Shannon sediment particle‐size diversity; percent sediment sand content	Positive relationship between sediment particle‐size diversity and peracarid biodiversity metrics; changes in sediment sand content will drive changes in peracarid abundance, biomass, biodiversity metrics, and assemblage structure	Complex relationship between sediment particle‐size diversity and peracarid biomass, no relationship between sediment particle‐size diversity and peracarid biodiversity metrics; significant relationship between sediment sand content and peracarid abundance, biodiversity metrics, and assemblage structure
Food availability	Surface chlorophyll a/POC concentration; seafloor POC concentration; sediment percent total carbon content; sediment percent organic carbon content	Unimodal relationship between food availability and peracarid biodiversity metrics; positive relationship between food availability and peracarid abundance and biomass; changing food availability will influence peracarid assemblage structure	Results contrast between two groupings—“total carbon” (surface POC, seafloor POC, and sediment total carbon content) and “organic carbon” (surface chlorophyll *a* and sediment organic carbon content) proxies. For “total carbon” proxies, high food availability alters peracarid assemblage structure, reduces abundance, biomass, and richness, but increases evenness and diversity. For “organic carbon” proxies, increasing food availability alters peracarid assemblage structure, increases abundance, reduces diversity and phylogenetic richness, and relates unimodally with evenness and functional richness. Results lend support toward a unimodal relationship between food availability and peracarid biodiversity
Current speed	Average seafloor current speed for year of sample collection; maximum seafloor current speed over 10 years prior to sample collection	Unimodal relationship between seafloor current speed and peracarid abundance, biomass, and biodiversity metrics; peracarid assemblage structure will vary with changes in seafloor current speed	Unimodal relationship between seafloor current speed and peracarid abundance and biodiversity metrics; peracarid assemblage structure varies with maximal decadal current speed
Physical disturbance (trawling intensity)	Trawling path density	Increasing trawling intensity will alter peracarid assemblage structure; negative relationship between trawling intensity and peracarid abundance, biomass and biodiversity metrics	Positive relationship between trawling intensity and peracarid abundance, and negative relationship with evenness and diversity; increasing trawling intensity alters peracarid assemblage structure
Temperature	Average seafloor temperature for year of sample collection; average seafloor temperature over 10 years prior to sample collection	Increasing temperature will alter peracarid assemblage structure and reduce peracarid abundance and biomass; unimodal relationship between seafloor temperature and peracarid biodiversity metrics	Increasing temperature alters peracarid assemblage structure and reduces peracarid biomass and diversity; positive relationship between 10‐year mean seafloor temperature and the phylogenetic richness of peracarid assemblages

### 1.2 Sediment characteristics

At small scales—centimeters to meters, sediment particle‐size diversity has been shown to correlate positively with macrofaunal and meiofaunal diversity (Etter & Grassle, 1992; Leduc, Rowden, Probert, et al., 2012; Pape, Bezerra, Jones, & Vanreusel, 2013). This relationship may reflect differences in particle‐size preference among benthic taxa (Etter & Grassle, 1992; Flach & Thomsen, 1998; Leduc, Rowden, Probert, et al., 2012). Average sediment particle size, which is closely related to near seabed current speed (McCave, Thornalley, & Hall, 2017), is also thought to influence deep‐sea macrobenthic assemblage characteristics. This is because it can influence a suite of secondary factors relevant to benthic communities, including changes in oxygen concentration with sediment depth, ease of burrow/tube construction, level of physical support provided, and identity of effective feeding strategies. As such, average sediment grain size may influence the morphological, physiological, and behavioral characteristics of benthic taxa present at a site (Johnson, 1971). For example, deposit‐feeding taxa, which are common constituents of benthic continental slope macrofauna (Etter & Grassle, 1992), can be highly selective for particular sediment particle sizes when feeding (Fenchel, 1975; Fenchel, Kofoed, & Lappalainen, 1975; Self & Jumars, 1988; Taghon, 1982), and hence, changes in sediment characteristics may drive turnover in benthic functional and taxonomic structure and diversity (Biernbaum, 1979; Cooper et al.., 2011). We hypothesize that sediment particle‐size diversity will be positively related to peracarid diversity and that changes in sediment average grain size will be associated with changes in peracarid diversity, abundance, biomass, and assemblage structure (Table 2).

### 1.3 Food availability

The rate, regularity, and quality of organic material being delivered to the deep seafloor are considered to be fundamental factors shaping deep‐sea benthic communities (Campanyà‐Llovet, Snelgrove, & Parrish, 2017; McClain, Allen, Tittensor, & Rex, 2012; Thiel, 1979). Food availability has been shown to have a controlling influence over benthic faunal abundance, biomass, diversity, body size, oxygen consumption, and assemblage composition (Corliss, Brown, Sun, & Showers, 2009; Gooday, Turley, & Allen, 1990; Johnson et al., 2007; Pilditch, Leduc, Nodder, Probert, & Bowden, 2015; Rex et al., 2006; Ruhl & Smith, 2004; Smith, Leo, Bernardino, Sweetman, & Arbizu, 2008; Smith, Ruhl, Kahru, Huffard, & Sherman, 2013; Woolley et al., 2016). Temporal changes in surface phytoplanktonic communities can have considerable influence over ecological processes in the deep sea, often with surprisingly little time lag (Billett, Bett, Reid, Boorman, & Priede, 2010; Graf, 1989; Johnson et al., 2007; Ruhl & Smith, 2004; Smith et al., 2006, 2013).

Relationships between food availability and organismal diversity have been argued to be either linear (positive or negative) or unimodal in form (Cusens, Wright, McBride, & Gillman, 2012; Grime, 1973; Mittelbach et al., 2001; Rosenzweig & Abramsky, 1993; Waide et al., 1999), and in the deep ocean, both of these forms have been reported (Levin & Dayton, 2009; Levin & Sibuet, 2012; McClain & Schlacher, 2015). For example, Lambshead, Tietjen, Ferrero, and Jensen (2000), Lambshead et al. (2002) reported a positive relationship between nematode species diversity and surface productivity in the North Atlantic and Pacific oceans at bathyal, abyssal, and hadal depths, while Tittensor, Rex, Stuart, McClain, and Smith (2011), Leduc, Rowden, Bowden, et al. (2012), and Jöst et al. (2019) reported a unimodal relationship between molluscan, nematode, and ostracod diversity, respectively, and food availability proxies at bathyal and abyssal depths. Levin et al. (2001) specified a unimodal relationship between food availability and taxon richness whereby at low food availability, diversity is depressed as a result of insufficient resources to support viable populations of species, whereas at high food availability, diversity is depressed because of a combination of reduced environmental heterogeneity and increased species dominance, resulting in a diversity maxima at intermediate levels of food availability. We hypothesize that peracarid diversity will be unimodally related to food availability, while peracarid abundance and biomass will be positively related to food availability. We hypothesize that changing food availability will be associated with changes in peracarid assemblage structure (Table 2).

### 1.4 Current speed

The relationship between near‐bottom current regimes and benthic faunal attributes is complex since water currents can physically influence fauna, as well as shape other aspects of their environment. In areas of relatively low current speed, rates of food delivery, as well as oxygen concentration, may be limiting to fauna, which can result in depressed faunal abundance and diversity (Levin et al., 2001). If current speed is moderate, however, faunal abundance and diversity may be enhanced because of increased delivery of organic material (Davies et al., 2009; Levin et al., 2001; Reidenauer & Thistle, 1985; Thistle, Yingst, & Fauchald, 1985), assuming that food delivery is not excessive. Moderate currents also promote the dispersal of fauna by the entrainment of larval and subadult individuals, while varied flow conditions can increase sediment heterogeneity (Aller, 1989). In combination, these factors can lead to enhanced alpha diversity. Under excessive current flow, however, faunal abundance and diversity may be depressed as a result of the erosion of surficial sediments (sometimes including their fauna), homogenization of sediment structures, and suppression of ecological succession (Levin et al., 2001). For example, Gage, Lamont, and Tyler (1995) and Gage (1997) reported polychaete diversity to be highest at tranquil sites on the Tagus Abyssal Plain and in the central North Pacific, lower at bathyal depths in the Rockall Trough and in the hydrodynamically active Sebutal Canyon, and much reduced at the abyssal HEBBLE site off Nova Scotia, which experiences the highest flow regimes. We hypothesize a unimodal relationship between seafloor current speed and peracarid abundance, biomass, and diversity, and that peracarid assemblage structure will vary with changes in seafloor current speed (Table 2).

### 1.5 Physical disturbance and bottom trawling

The “Intermediate Disturbance Hypothesis” (Connell, 1978) proposes that the relationship between disturbance frequency/magnitude and faunal diversity is unimodal. At low levels of disturbance, diversity is reduced as a result of low environmental heterogeneity and high faunal dominance (Connell, 1978; Hobbs & Huenneke, 1992). Periodic physical disturbance of intermediate intensity (for instance, as a result of the activity of mobile megafauna, or episodically raised current speeds) can, however, create a mosaic of habitats at varying stages of community succession (Gage, 1996; Huston, 1979; Kukert & Smith, 1992), enhancing diversity. As disturbance frequency and magnitude increase further, environmental conditions may exceed the physiological tolerances of the majority of species, resulting in a depauperate community (Connell, 1978; Hobbs & Huenneke, 1992; Levin et al., 2001). For example, polychaete diversity in North Atlantic bathyal and abyssal sites has been shown to peak at intermediate disturbance levels (Cosson‐Sarradin, Sibuet, Paterson, & Vangriesheim, 1998; Paterson & Lambshead, 1995).

Commercial bottom trawling for demersal fish represents a substantial form of disturbance to continental slope benthic communities and has been associated with considerable incidental damage and/or removal as bycatch of benthic megafauna (Clark et al., 2016). Deep‐sea bottom trawling can drive habitat modification (such as damage to “Vulnerable Marine Ecosystems”) and changes to benthic epifaunal assemblage structure, with reductions in the biomass and diversity of associated benthic assemblages (Clark et al., 2016; Clark & Rowden, 2009; Hall‐Spencer, 2002; Koslow & Gowlet‐Holmes, 1998; Koslow et al., 2001; Pusceddu et al., 2014). Further, the physical action of bottom trawling can lead to smoothing of the seafloor over large spatial scales (Clark & Rowden, 2009; Puig et al., 2012), reduce the bioavailability of carbon (Pusceddu et al., 2014, 2005), and alter the grain‐size characteristics of sediments through resuspension (Martín, Puig, Masqué, Palanques, & Sánchez‐Gómez, 2014). The net result of these impacts is an overall reduction in seafloor heterogeneity and organic carbon availability with increasing trawling pressure (Clark & Rowden, 2009; Rogers, Clark, Hall‐Spencer, & Gjerde, 2008). We hypothesize that increasing trawling intensity will be associated with a change in the structure of peracarid assemblages and a reduction in their abundance, biomass, and diversity (Table 2).

### 1.6 Temperature

Temperature represents a fundamental factor shaping biological communities, with demonstrable influence over faunal diversity, distributions, recruitment, growth rate, and species interactions in the marine environment (Ashford, Davies, & Jones, 2014; Danovaro, Dell'Anno, & Pusceddu, 2004; Poloczanska et al., 2013; Tittensor et al., 2010; Woolley et al., 2016; Yasuhara & Danovaro, 2016; Yasuhara, Tittensor, Hillebrand, & Worm, 2017). In the deep sea, temperature has been shown to have a significant influence over the ecological characteristics of benthic assemblages, including species diversity, biomass, and assemblage composition (Barrio Froján, 2012; Jöst et al., 2019; Yasuhara, Cronin, Menocal, Okahashi, & Linsley, 2008; Yasuhara & Danovaro, 2016; Yasuhara, Okahashi, Cronin, Rasmussen, & Hunt, 2014).

Crucially, the thermal stability of the deep sea compared with other biomes may mean that deep‐water species are less tolerant of changing temperature than shallow‐water/ terrestrial species (Howes, Joos, Eakin, & Gattuso, 2015; McCauley et al., 2015; Tewksbury, Huey, & Deutsch, 2008; Yasuhara, Hunt, Cronin, & Okahashi, 2009). Thus, even small changes in temperature as a result of climate change may impact species' distributions and hence deep‐sea diversity patterns. Increasing deep‐sea temperatures will also increase faunal metabolic rates (Brown, Gillooly, Allen, Savage, & West, 2004; Hochachka & Somero, 2002; McClain et al., 2012; Seibel & Drazen, 2007). This, coupled with potentially reduced delivery of surface primary productivity to the seafloor, may negatively impact faunal biomass (Jones et al., 2014; Rogers, 2015; Sweetman et al., 2017). However, the potential impacts of rising temperature on deep‐sea benthos await investigation on a large scale (Sweetman et al., 2017; Yasuhara & Danovaro, 2016). The association between temperature and faunal diversity was not considered by Levin et al. (2001), but based on the investigation of both contemporary and ancient seafloor assemblages, Yasuhara and Danovaro (2016) proposed a unimodal relationship between temperature and diversity. We hypothesize that higher temperatures will be associated with altered peracarid assemblage structure and reduced peracarid abundance and biomass. We hypothesize that the relationship between seafloor temperature and peracarid diversity will be unimodal in form (Table 2).

## 2 METHODS

### 2.1 Faunal sample acquisition and processing

**Figure 2:**
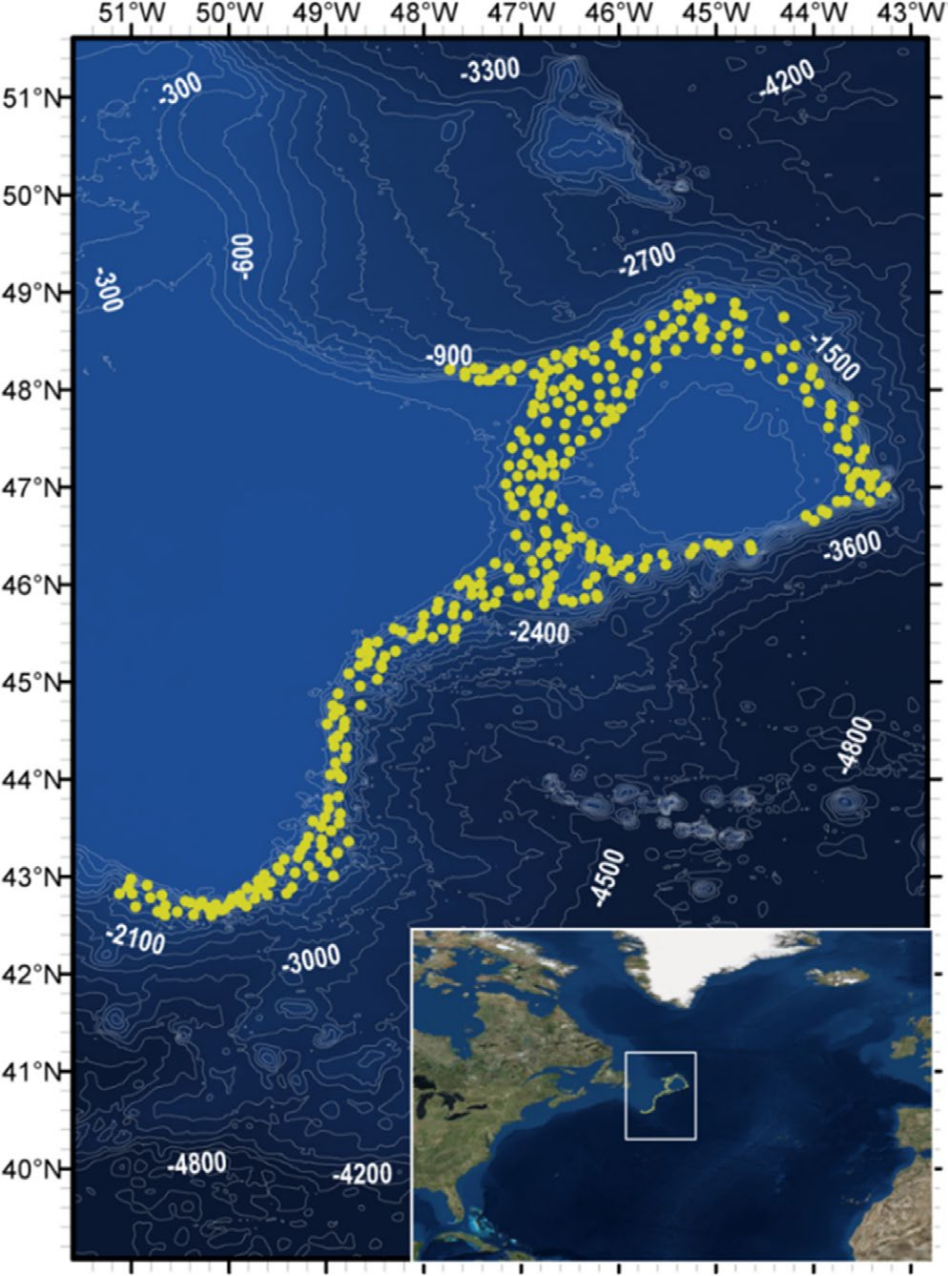
Sampling locations (yellow dots; *n* = 312) and bathymetry of sampling region (darker areas are of greater water depth) in the Northwest Atlantic Ocean (300‐meter‐depth contours; SRTM30 bathymetric data). Inset map places sampling area (white box) in a global context (Satellite imagery courtesy of ESRI World Imagery)

Peracarid crustacean assemblages were sampled by means of 312 box cores (USNEL type, area 0.25 m^2^) collected over spring and summer in 2009 and 2010 from the high seas of the Northwest Atlantic (Flemish Pass, the slopes of the Flemish Cap and the continental slopes of the Grand Banks; depth range: 582–2,294 m; Figure 2). These cores were collected as part of the international “NAFO (Northwest Atlantic Fisheries Organization) potential vulnerable marine ecosystems – impacts of deep‐sea fisheries” (NEREIDA) program (see Ashford et al. (2018) for complete details of sample processing).

A total of 20,245 individual peracarids were identified to 177 genera and 74 families. For each sample, peracarid abundance (total number of individuals) was enumerated and total wet biomass was recorded to an accuracy of 0.0001 g. Taxon richness was calculated as the number of genera per sample. Rao's quadratic entropy and Shannon's diversity index were calculated as metrics of taxon diversity for each sample using the packages “SYNCSA 1.3.2” (Debastiani, 2015) and “vegan 2.0‐9” (Oksanen et al., 2013), respectively, in R 3.0.2 (R Core Team, 2013). Pielou's Index was calculated as a metric of taxon evenness using R.

Assemblage phylogenetic diversity was calculated based on the supertree of Peracarida presented by Ashford et al. (2018) and genus‐level assemblage composition data for each sample. The metrics “Phylogenetic Species Richness” (PSR) and “Phylogenetic Species Evenness” (PSE), developed by Helmus, Bland, Williams, and Ives (2007), were calculated in R 3.0.2 (R Core Team, 2013) using the package “picante 1.6‐2” (Kembel et al., 2014). PSR is calculated by multiplying “Phylogenetic Species Variability” (PSV), a measure of the overall phylogenetic relatedness of taxa in a sample, by the taxon richness of that sample. PSE is calculated by modifying PSV to incorporate the relative abundance of taxa in a sample. These metrics facilitate the incorporation of phylogenetic information into measures of taxon richness and taxon evenness under a single theoretical framework (Ashford et al., 2018; Helmus et al., 2007; Table 1). See Helmus et al. (2007) for a full mathematical explanation and exploration of these metrics. As analyses undertaken here are not at the species level, these metrics are referred to simply as “Phylogenetic Richness” (PR) and “Phylogenetic Evenness” (PE) henceforth.

Functional diversity was calculated based on the functional dendrogram of Peracarida presented by Ashford et al. (2018) and family‐level composition data for each sample. To promote consistency in methodology (and since functional dendrograms and phylogenies are analogous in form [Petchey & Gaston, 2007]), we calculated functional richness and functional evenness using the metrics “Phylogenetic Species Richness” (PSR) and “Phylogenetic Species Evenness” (PSE; Helmus et al., 2007) utilizing the R package “picante 1.6‐2” (Kembel et al., 2014). In this case, these metrics facilitate the incorporation of *functional* information into measures of taxon richness and taxon evenness. To avoid confusion with the measures of *phylogenetic* diversity investigated, functional diversity measures are referred to herein as “Functional Richness” (FR) and “Functional Evenness” (FE; Table 1).

### 2.2 Quantifying the physical environment

Environmental parameters for which information was available included depth, slope, eastness and northness, seafloor roughness (225 × 225 m analysis window), standard deviation of bathymetry values (225 × 225 m analysis window), seafloor rugosity (375 × 375 m analysis window and 1,875 × 1,875 m analysis window), Bathymetric Position Index (BPI) over a range of radii (outer radii of 1,875, 3,750, 5,625, 7,500, 9,375, and 11,250 m, inner radius of 75 m in all cases), geological environment category (12 categories—see Data S2), sample sediment percent clay/silt/sand, sample sediment particle‐size diversity, sample sediment total/organic/inorganic carbon content, mean sea‐surface chlorophyll *a* and particulate organic carbon (POC) concentrations over sample collection year and previous year, mean delivery of POC to seafloor depth over sample collection year and previous year, mean seafloor temperature/salinity/current speed for sample collection year, maximum/minimum/mean seafloor temperature and current speed for decade prior to sample collection, and total bottom trawling intensity between 2008 and 2012 within 1, 3, and 5 km radii of sample locations (Table 3). Sample collection date (year and month) and crew identity were also recorded. In order to enable relation of peracarid assemblage metrics to their physical environment, these environmental data were collated into a dataset (Data S2) summarizing the values of parameters at sampling locations. Ashford et al. (2018) includes detailed methodology relating to the calculation of these environmental parameters at sampling locations.

**Table 3:** Environmental variables retained for analysis following variance inflation factor variable selection

Environmental facet	Representing variable	Origin
Geological environment	Geological environment (categorical)	Categorized based on cruise multibeam data
Seafloor topography	Seafloor rugosity—value for a 1,875 × 1,875 m analysis window around sample location	Calculated from cruise multibeam data
	Seafloor roughness—value for a 225 × 225 m analysis window around sample location	Calculated from cruise multibeam data
	Bathymetric Position Index—value for 9,375 m radius analysis window around sample location	Calculated from cruise multibeam data
Sediment grain size	Percent sand content in top 2 cm of sample	Quantified from box core subsamples
	Shannon sediment particle‐size diversity for top 2 cm of sample	Calculated from sample particle‐size analysis data
Surface productivity	Surface chlorophyll *a*—MODIS average for year of sample collection plus previous year (mg/m^3^)	Downloaded from Giovanni ocean color radiometry online data system (https://giovanni.gsfc.nasa.gov/giovanni/)
	Surface particulate organic carbon—MODIS average for year of sample collection plus previous year (mg/m^3^)	Downloaded from Giovanni ocean color radiometry online data system (https://giovanni.gsfc.nasa.gov/giovanni/)
Energy availability at seafloor	Particulate organic carbon at seafloor—Lutz, Dunbar, and Caldeira (2002) Sargasso Sea equation (not radionuclide corrected) applied to MODIS surface particulate organic carbon average for year of sample collection plus previous year (mg/m^3^)	Calculated from surface particulate organic carbon data
	Percent total carbon content in top 2 cm of sample	Quantified from box core subsamples
	Percent organic carbon content in top 2 cm of sample	Quantified from box core subsamples
Current speed	Seafloor absolute current speed—average for year of sample collection (m/s)	Extracted from a modeled data layer for the study area
	Seafloor maximum current speed—maximum value over 10 years prior to sample collection (m/s)	Extracted from a modeled data layer for the study area
Trawling intensity	Trawl density—total trawl length per km^2^ of seafloor between 2008 and 2012 within a 3 km radius of the sample location (Log_10_)	Calculated from vessel monitoring system data
Temperature	Seafloor temperature—average for year of sample collection (°C)	Extracted from a modeled data layer for the study area
	Seafloor temperature— average for 10 years prior to sample collection (°C)	Extracted from a modeled data layer for the study area
Annual and seasonal variation	Collection year—sample collection year (categorical)	NA
	Collection month—sample collection month (categorical)	NA
Variation in collection procedure	Crew identity at time of sample collection (categorical)	NA

Note

See Ashford et al. (2018) for full details of environmental variables.

### 2.3 Statistical analyses

#### 2.3.1 Investigation of multicollinearity among independent variables

Missing values in environmental data (0.42% of values) were replaced with the arithmetic mean for that variable. Highly correlated variables were removed (since high multicollinearity can have severe effects on the estimation of model parameters [Gunst & Webster, 1975]) following a two‐step process. First, where a set of variables at different spatial scales were available for a single facet of environmental variation (e.g., trawling intensity data were available for 1, 3, and 5 km radii), the variable in that set with the highest variance inflation factor (VIF) was selected to represent that particular environmental facet since this variable best captured the information present in the alternative variables. Secondly, a round of VIF calculations was undertaken among all selected variables from the first step. Variables were removed from the analysis in a stepwise manner (those with highest VIF first) until all variables had a VIF value <5 (Stine, 1995). The resulting dataset contains 19 variables describing the physical environment (Table 3). VIF calculations were undertaken in R 3.0.2 using the package “HH 3.1‐32” (Heiberger, 2016). See Table S1 for a correlation matrix of the continuous environmental variables retained for analysis.

#### 2.3.2 Construction of Generalized Additive Models

Because initial data exploration revealed a high frequency of nonlinear relationships between dependent and independent variables, we constructed Generalized Additive Models (GAMs) in R 3.0.2 (R Core Team, 2013) using the package “mgcv 1.7‐26” (Wood, 2013a). Individual multivariate GAMs described the relationships between each univariate “biodiversity” metric (i.e., functional diversity and Shannon diversity [Table 1]) and the independent physical environmental variables (Table 3). Prior to analysis, trawling intensity (3 km radius) was transformed (Log_10_ (*x* + 1)) to reduce extreme skew in the data. Original measurement scales were retained for all other variables because data distributions were deemed acceptable and in order to simplify the interpretation of model outputs. Samples with no peracarids, and sample number 341 (with only one peracarid genus present), were removed from the dataset prior to analysis.

Initial GAMs consisted of all variables contained within Table 3, with smoothers added to all continuous variables. We selected the most appropriate error distributions and link functions for the models (Table S2) by consideration of the Akaike information criterion (AIC; Akaike, 1974) and model performance diagnostics. Acceptable satisfaction of model assumptions was investigated using the *gam.check* function of the package “mgcv 1.7‐26” (Wood, 2013a). Where the biological metric was categorical in form (“Abundance” and “Taxon Richness”), overdispersion was checked for by calculation of the dispersion parameter “theta” (where theta = residual deviance/residual degrees of freedom) and corrected for as appropriate (Table S2). A penalized thin‐plate regression spline was used as the smoothing function, and smoothing parameters were optimized automatically on the basis of the generalized cross‐validation criterion (Wood, 2013a).

Spatial autocorrelation in model residuals was investigated by calculating Moran's I (Moran, 1950) in R using the package “ape 3.2” (Paradis, Claude, & Strimmer, 2004). We found no evidence of spatial autocorrelation in any of the models constructed.

We refined the explanatory terms included in each GAM (Table S2) backward from the full model by stepwise selection after considering independent variable *p*‐values and model AIC until a minimum AIC value was reached. Because *p*‐values derived from GAMs for smoothed terms are approximate (Wood, 2013a, 2013b), results are presented for smoothed model terms where *p* > .05 (but <0.1), and these are referred to as “weak,” “possible,” or “marginal.” Nonsignificant relationships (*p* > .1) are not reported unless explicitly stated.

#### 2.3.3 Relation of assemblage structure to physical variables

The relationship between peracarid assemblage structure (identities and relative abundances of genera in each sample) and the physical environment was investigated by constrained analysis of principal coordinates (CAP; Anderson & Willis, 2003) based on Bray–Curtis dissimilarity (Bray & Curtis, 1957), followed by backward model refinement based on *p*‐values using the function *ordistep* in the R package “vegan 2.0‐9” (Oksanen et al., 2013; 10,000 permutations, *p*‐in = .05, *p*‐out = .1). Constrained ordination methods explicitly relate a matrix of response variables to a selection of predictor variables. Such an approach facilitates the direct relation of assemblage structure to the physical environment within a single‐analysis framework (Anderson & Willis, 2003). CAP has the advantage over other canonical analysis techniques that any distance or dissimilarity measure can be used (Anderson & Willis, 2003).

## 3 RESULTS

### 3.1 Topography

Peracarid abundance was found to relate significantly with the geological environment from which a box core was collected (*p* = .031), with abundance being lowest on “steep flanks” relative to other environments (*p* = .048). Peracarid taxon assemblage structure was also significantly related to geological environment (*p* < .001).

**Figure A1:**
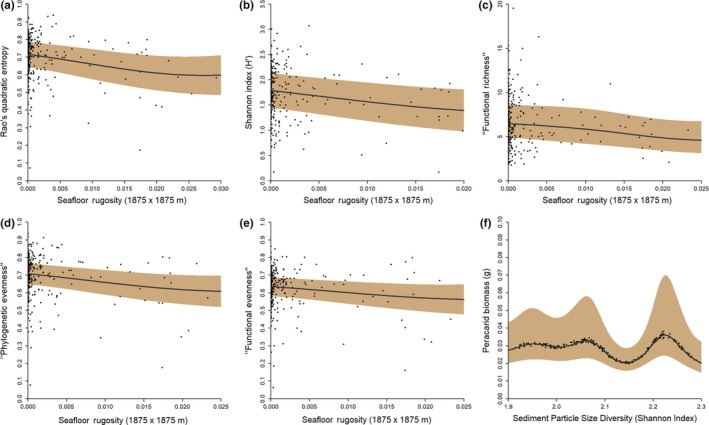
Partial relationships between seafloor rugosity/sediment particle‐size diversity (PSD) at sampling locations (*n* = 312) in the Northwest Atlantic Ocean and different metrics of the biodiversity of peracarid assemblages sampled. Solid lines are smoothed lines of best fit as determined by multivariate generalized additive modeling. Colored bands are 95% confidence intervals. (a) Rugosity/Rao's quadratic entropy; (b) rugosity/Shannon Index; (c) rugosity/functional richness; (d) rugosity/phylogenetic evenness; (e) rugosity/functional evenness; (f) PSD/biomass

A negative relationship was observed between seafloor topographic heterogeneity (“rugosity”; 1,875 × 1,875 m analysis window; Figure A1) and phylogenetic evenness (PE; *p* = .003), functional evenness (FE; *p* = .021), Rao's quadratic entropy (*p* = .002), Shannon diversity (*p* = .003), and possibly functional richness (FR; *p* = .072). Further, seafloor “rugosity” was found to relate significantly with peracarid taxon assemblage structure (*p* = .002).

**Figure A2:**
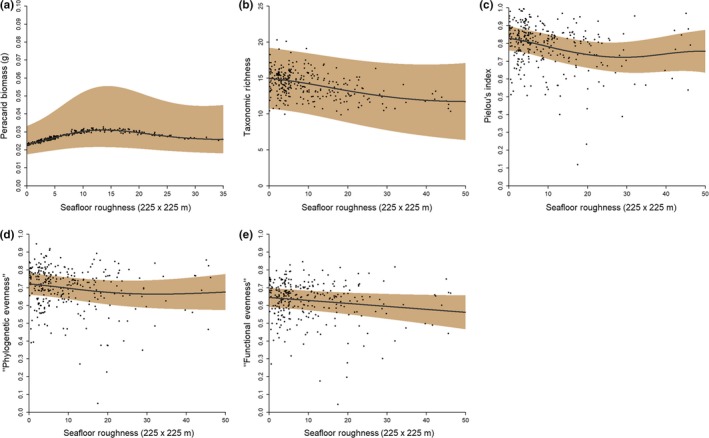
Partial relationships between seafloor roughness at sampling locations (*n* = 312) in the Northwest Atlantic Ocean and different metrics of the biodiversity of peracarid assemblages sampled. Solid lines are smoothed lines of best fit as determined by multivariate generalized additive modeling. Colored bands are 95% confidence intervals. (a) roughness/biomass; (b) roughness/taxonomic richness; (c) roughness/Pielou's Index; (d) roughness/phylogenetic evenness; (e) roughness/functional evenness

Seafloor “roughness” (225 × 225 m analysis window; Figure A2) was found to relate negatively with PE (*p* = .042), possibly FE (*p* = .052), and taxon richness (*p* = .016). However, the relationship between “roughness” and peracarid biomass was shallow unimodal (*p* = .027), while Pielou's Index was found to vary in a U‐shaped manner with “roughness” (*p* = .002).

**Figure 3:**
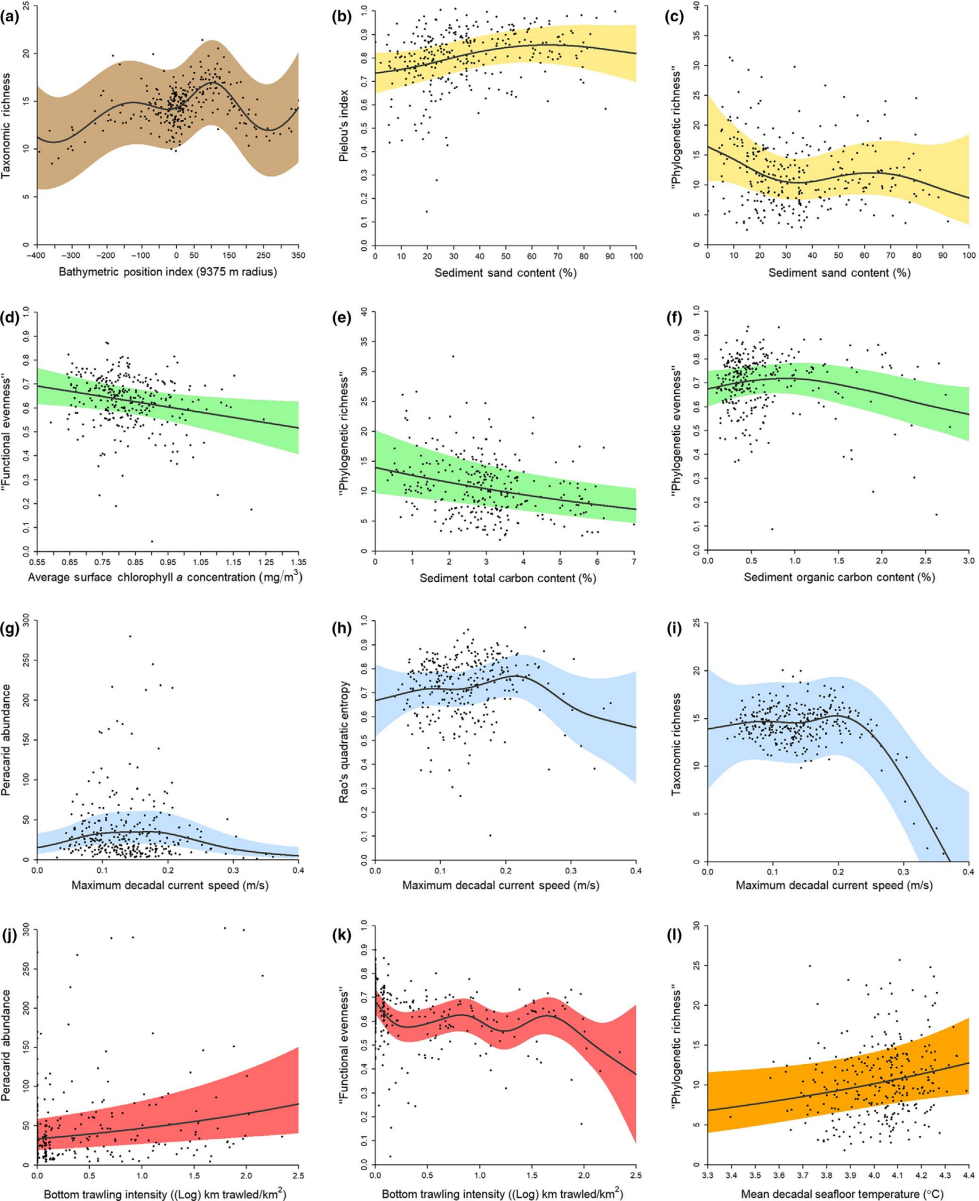
Partial relationships between environmental conditions at sampling locations (*n* = 312) in the Northwest Atlantic Ocean and different metrics of the biodiversity of peracarid assemblages sampled. Solid lines are smoothed lines of best fit as determined by multivariate generalized additive modeling. Colored bands are 95% confidence intervals. (a) Bathymetric Position Index (brown)/taxonomic richness; (b) sediment sand content (yellow)/phylogenetic richness; (c) sediment sand content/Pielou's index; (d) sediment total carbon content (food availability: green)/phylogenetic richness; (e) sediment organic carbon content/phylogenetic evenness; (f) surface chlorophyll *a* concentration/functional evenness; (g) maximum decadal current speed (blue)/abundance; (h) maximum decadal current speed/Rao's quadratic entropy; (i) maximum decadal current speed/taxonomic richness; (j) bottom trawling intensity (red)/abundance; (k) bottom trawling intensity/functional evenness; (l) mean decadal seafloor temperature (orange)/phylogenetic richness

**Figure A3:**
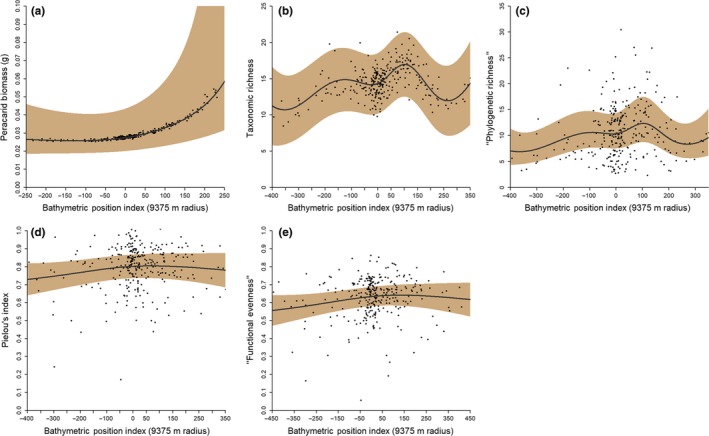
Partial relationships between Bathymetric Position Index (BPI) at sampling locations (*n* = 312) in the Northwest Atlantic Ocean and different metrics of the biodiversity of peracarid assemblages sampled. Solid lines are smoothed lines of best fit as determined by multivariate generalized additive modeling. Colored bands are 95% confidence intervals. (a) BPI/biomass; (b) BPI/taxonomic richness; (c) BPI/phylogenetic richness; (d) BPI/Pielou's Index; (e) BPI/functional evenness

Bathymetric Position Index (BPI), a measure of the elevation of a focal point relative to its surroundings (here defined by a 9,375‐m outer radius window and a 75‐m inner radius window; Figure A3), was found to relate significantly with peracarid biomass (*p* < .001). Faunal biomass was relatively elevated in samples situated topographically higher than surrounding areas (i.e., on ridges). A shallow unimodal relationship was observed between BPI and Pielou's Index (*p* = .048) and FE (*p* = .013), with evenness peaking at locations that are moderately elevated relative to the surrounding region. Peracarid taxon and phylogenetic richness were found to be maximal at sampling locations that are relatively elevated compared with their surroundings (*p* = .004; *p* = .024, respectively; Figure 3a). BPI was also found to relate significantly with peracarid taxon assemblage structure (*p* < .001).

### 3.2 Sediment characteristics

Sediment particle‐size diversity (PSD) was significantly related only to peracarid biomass (*p* = .002). This relationship was complex in form, but highest peracarid biomass was associated with relatively high values of PSD (Figure A1f).

**Figure A4:**
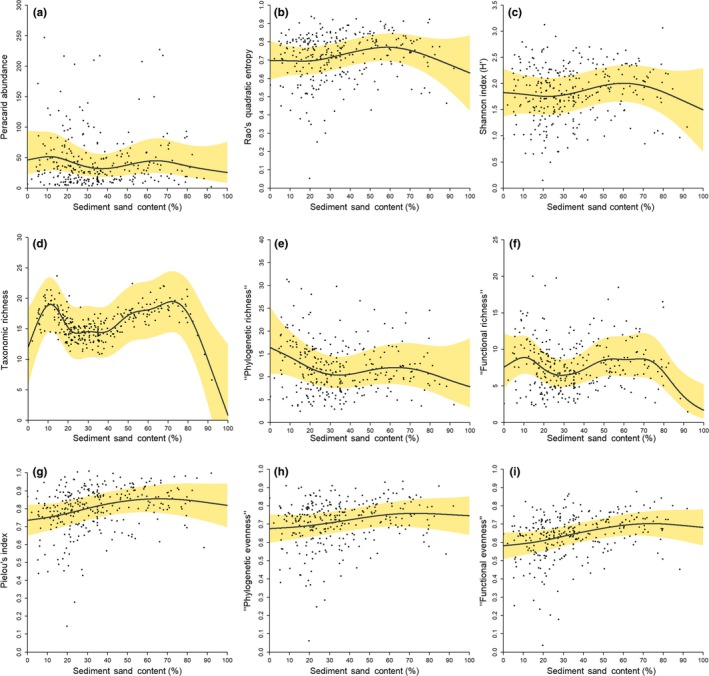
Partial relationships between sediment sand content (%) at sampling locations (*n* = 312) in the Northwest Atlantic Ocean and different metrics of the biodiversity of peracarid assemblages sampled. Solid lines are smoothed lines of best fit as determined by multivariate generalized additive modeling. Colored bands are 95% confidence intervals. (a) sand percent/peracarid abundance; (b) sand percent/Rao's quadratic entropy; (c) sand percent/Shannon Index; (d) sand percent/taxonomic richness; (e) sand percent/phylogenetic richness; (f) sand percent/functional richness; (g) sand percent/Pielou's Index; (h) sand percent/phylogenetic evenness; (i) sand percent/functional evenness

The percent sand content of box core surficial sediments (Figure A4) was found to relate significantly with peracarid abundance (*p* = .005), with abundance peaking at both low (~0%–15%) and high (~60%–75%) proportions of sand. Sand content was also found to correlate with an increase in FE (*p* < .001) and PE (*p* = .009), while a unimodal relationship was observed between sand content and Pielou's Index (*p* < .001; Figure 3b), Rao's quadratic entropy (*p* = .031), and possibly Shannon diversity (*p* = .062). These diversity and evenness metrics peaked between ~55% and 70% sand content. The sediment characteristics of each sample were also found to be correlated with taxonomic, phylogenetic, and functional metrics of richness. The relationships between percent sand content and FR (*p* = .002) and taxon richness (*p* < .001) are both double‐peaked, with maximal functional and taxonomic richness of peracarids at both lower (~8%–13%) and higher (~48%–78%) proportions of sand. Values of PR also peak at relatively low and high proportions of sand (*p* = .019), with PR values being slightly depressed between ~25% and ~45% sand content (Figure 3c). Percent sand content of sample sediments was found to relate significantly with peracarid taxon assemblage structure (*p* < .001).

### 3.3 Food availability

**Figure A5:**
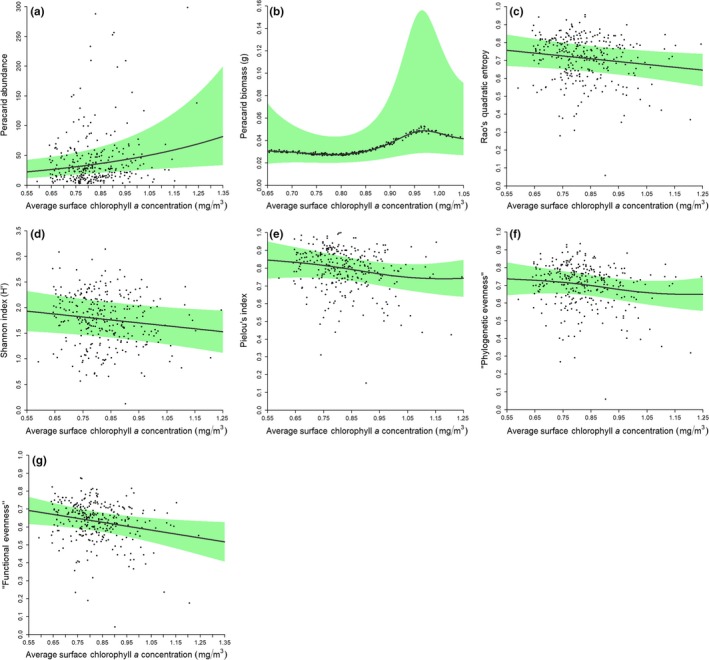
Partial relationships between surface chlorophyll *a* concentration (mg/m^3^) at sampling locations (*n* = 312) in the Northwest Atlantic Ocean and different metrics of the biodiversity of peracarid assemblages sampled. Solid lines are smoothed lines of best fit as determined by multivariate generalized additive modeling. Colored bands are 95% confidence intervals. (a) Chlorophyll *a*/peracarid abundance; (b) chlorophyll *a*/peracarid biomass; (c) chlorophyll *a*/Rao's quadratic entropy; (d) chlorophyll *a*/Shannon Index; (e) chlorophyll *a*/Pielou's Index; (f) chlorophyll *a*/phylogenetic evenness; (g) chlorophyll *a*/functional evenness

A number of complementary metrics of food availability to macrofauna were assessed. Average sea‐surface chlorophyll *a* concentration (Figure A5) was found to be positively correlated with peracarid abundance (*p* = .011) and biomass (*p* = .003), but negatively correlated with FE (*p* = .023; Figure 3d), PE (*p* = .007), Pielou's Index (*p* = .013), Rao's quadratic entropy (*p* = .019), and Shannon diversity (*p* = .011). Sea‐surface chlorophyll *a* concentration was also found to relate significantly with peracarid taxon assemblage structure (*p* < .001).

**Figure A6:**
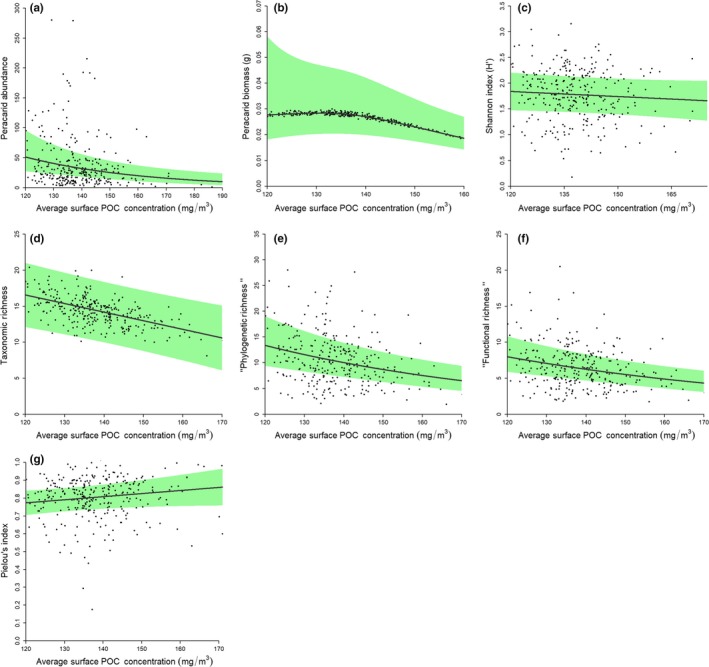
Partial relationships between surface particulate organic carbon (POC) concentration (mg/m^3^) at sampling locations (*n* = 312) in the Northwest Atlantic Ocean and different metrics of the biodiversity of peracarid assemblages sampled. Solid lines are smoothed lines of best fit as determined by multivariate generalized additive modeling. Colored bands are 95% confidence intervals. (a) Surface POC/peracarid abundance; (b) surface POC/peracarid biomass; (c) surface POC/Shannon Index; (d) surface POC/taxonomic richness; (e) surface POC/phylogenetic richness; (f) surface POC/functional richness; (g) surface POC/Pielou's Index

In contrast, average sea‐surface POC concentration values (Figure A6) were negatively correlated with peracarid abundance (*p* < .001), biomass (*p* = .005), the richness of peracarid genera (*p* < .001), PR (*p* < .001), FR (*p* < .001), and possibly Shannon diversity (*p* = .056). However, Pielou's Index showed a possible positive relationship with surface POC concentration values (*p* = .089). Sea‐surface POC concentration values were found to relate significantly with peracarid taxon assemblage structure (*p* < .001).

**Figure A7:**
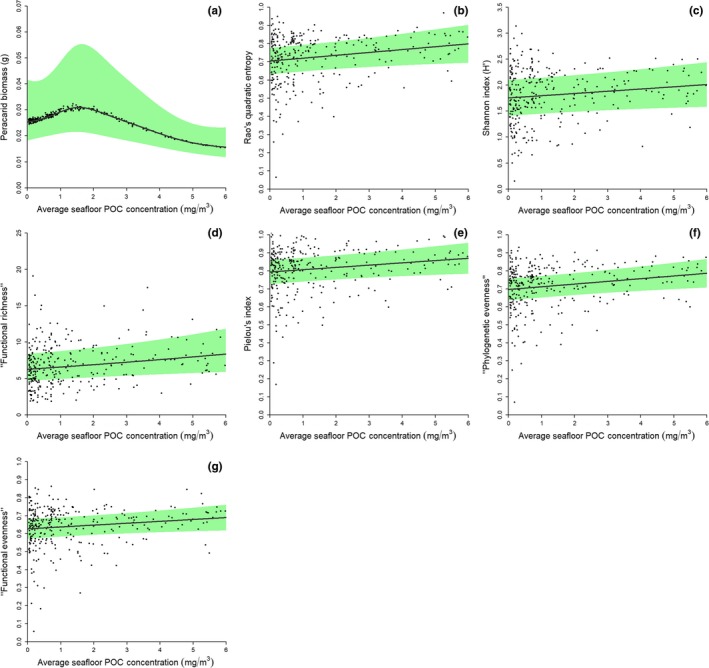
Partial relationships between seafloor particulate organic carbon (POC) concentration (mg/m^3^) at sampling locations (*n* = 312) in the NW Atlantic Ocean and different metrics of the biodiversity of peracarid assemblages sampled. Solid lines are smoothed lines of best fit as determined by multivariate generalized additive modeling. Colored bands are 95% confidence intervals. (a) Seafloor POC/peracarid biomass; (b) seafloor POC/Rao's quadratic entropy; (c) seafloor POC/Shannon Index; (d) seafloor POC/functional richness; (e) seafloor POC/Pielou's Index; (f) seafloor POC/phylogenetic evenness; (g) seafloor POC/functional evenness

Average seafloor POC concentration values (Figure A7) were unimodally related to peracarid biomass (*p* < .001). In contrast, positive relationships were observed between seafloor POC concentration and FR (*p* = .021), Pielou's Index (*p* = .025), PE (*p *= .004), FE (*p* = .041), Rao's quadratic entropy (*p* = .025), and Shannon diversity (*p* = .035). Further, seafloor POC concentration values related significantly with peracarid taxon assemblage structure (*p* < .001).

**Figure A8:**
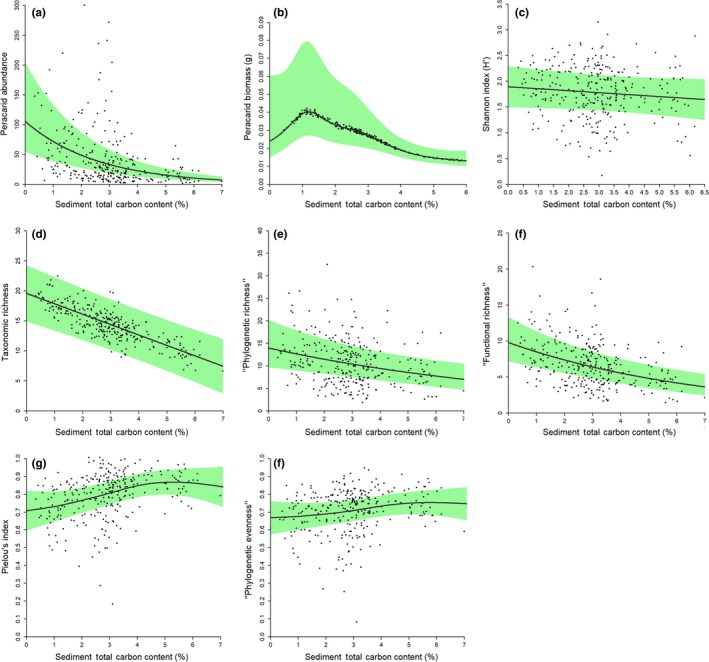
Partial relationships between sediment total carbon content (%) at sampling locations (*n* = 312) in the Northwest Atlantic Ocean and different metrics of the biodiversity of peracarid assemblages sampled. Solid lines are smoothed lines of best fit as determined by multivariate generalized additive modeling. Colored bands are 95% confidence intervals. (a) Total carbon/peracarid abundance; (b) total carbon/peracarid biomass; (c) total carbon/Shannon Index; (d) total carbon/taxonomic richness; (e) total carbon/phylogenetic richness; (f) total carbon/functional richness; (g) total carbon/Pielou's Index; (h) total carbon/phylogenetic evenness

Sediment total carbon content per sample (Figure A8) was negatively related to peracarid abundance (*p* < .001), PR (*p* = .001; Figure 3e), FR (*p* < .001), taxon richness (*p* < .001), and possibly Shannon diversity (*p* = .099). However, biomass was found to relate unimodally with sediment total carbon content (*p* < .001), and a positive relationship with PE (*p* = .015) and Pielou's Index (*p* < .001) was identified. Sediment total carbon content was found to relate significantly with peracarid taxon assemblage structure (*p* < .001).

**Figure A9:**
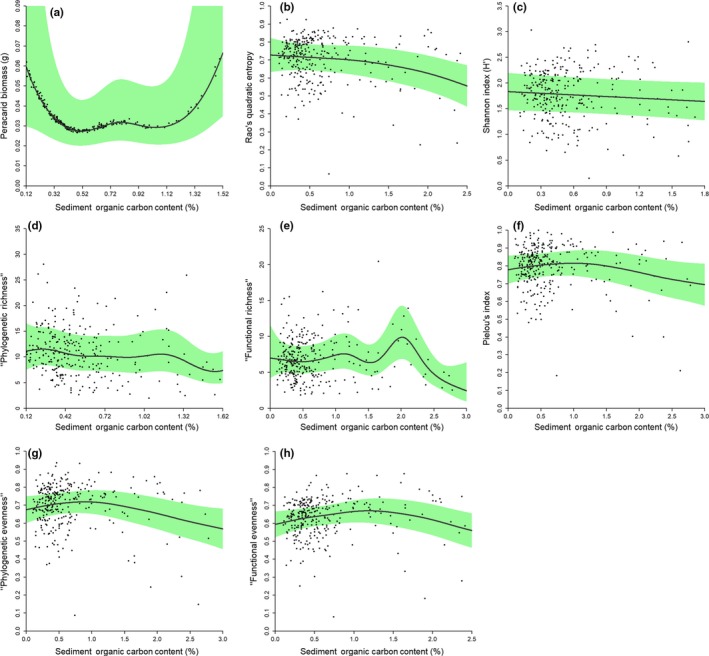
Partial relationships between sediment organic carbon content (%) at sampling locations (*n* = 312) in the NW Atlantic Ocean and different metrics of the biodiversity of peracarid assemblages sampled. Solid lines are smoothed lines of best fit as determined by multivariate generalized additive modeling. Colored bands are 95% confidence intervals. (a) Organic carbon/peracarid biomass; (b) organic carbon/Rao's quadratic entropy; (c) organic carbon/Shannon Index; (d) organic carbon/phylogenetic richness; (e) organic carbon/functional richness; (f) organic carbon/Pielou's Index; (g) organic carbon/phylogenetic evenness; (h) organic carbon/functional evenness

A significant U‐shaped relationship was found between sediment organic carbon content (Figure A9) and peracarid biomass (*p* < .001), while sediment organic carbon content and FR were related in a weakly unimodal fashion (*p* = .024), with FR peaking between ~1.7% and 2.3% organic carbon content. A unimodal relationship was observed between sediment organic carbon content and FE (*p* = .003), PE (*p* = .005; Figure 3f), and Pielou's Index (*p* = .019), all peaking at approximately ~0.5%–1.5% organic carbon. PR (*p* = .016), Rao's quadratic entropy (*p* = .002), and possibly Shannon diversity (*p* = .058) were found to relate negatively with sediment organic carbon content.

### 3.4 Current speed

**Figure A10:**
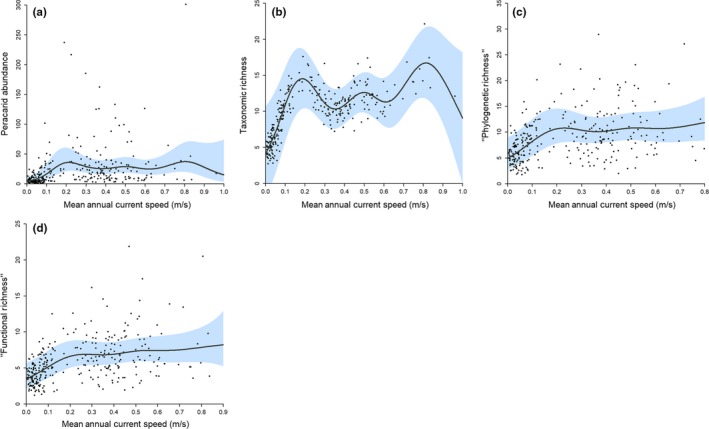
Partial relationships between mean annual current speed (m/s) at sampling locations (*n* = 312) in the Northwest Atlantic Ocean and different metrics of the biodiversity of peracarid assemblages sampled. Solid lines are smoothed lines of best fit as determined by multivariate generalized additive modeling. Colored bands are 95% confidence intervals. (a) Mean current speed/peracarid abundance; (b) mean current speed/taxonomic richness; (c) mean current speed/phylogenetic richness; (d) mean current speed/phylogenetic richness

Peracarid abundance varied significantly with the average seafloor current speed for the year of sample collection (Figure A10; *p* < .001), with faunal abundance rising sharply with increasing current speed to a peak at ~0.18 m/s, then dropping, and plateauing as current speed increases further. Average annual current speed at sample locations was found to be positively correlated with PR (*p* = .046) and FR (*p* = .043), with richness rising rapidly as current speed increases to ~0.20–0.25 m/s, and then declining slightly before increasing at a much‐reduced rate thereafter. Taxon richness was also found to be positively associated with average annual current speed at sample locations (*p* < .001), this relationship being undulating but positive overall in form.

**Figure A11:**
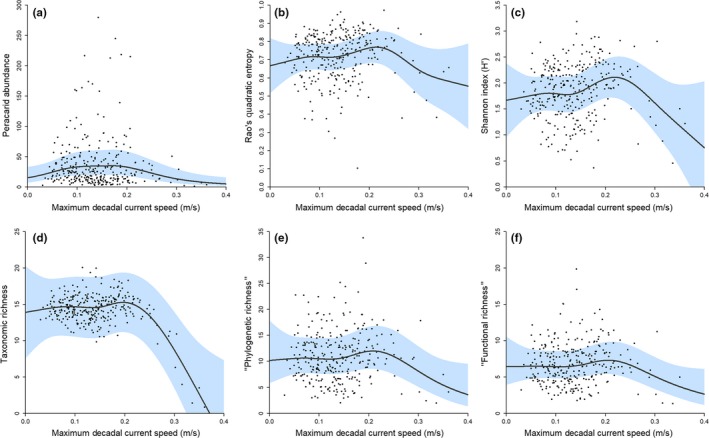
Partial relationships between maximal decadal current speed (m/s) at sampling locations (*n* = 312) in the Northwest Atlantic Ocean and different metrics of the biodiversity of peracarid assemblages sampled. Solid lines are smoothed lines of best fit as determined by multivariate generalized additive modeling. Colored bands are 95% confidence intervals. (a) Maximum current speed/peracarid abundance; (b) maximum current speed/Rao's quadratic entropy; (c) maximum current speed/Shannon Index; (d) maximum current speed/taxonomic richness; (e) maximum current speed/phylogenetic richness; (f) maximum current speed/functional richness

A significant unimodal relationship was found between maximal decadal current speed at sample locations (Figure A11) and peracarid abundance (*p* < .001). Peracarid abundance was found to peak at current speeds between ~0.10 m/s and 0.2 m/s (Figure 3g). Rao's quadratic entropy (*p* = .016) and Shannon diversity (*p* < .001) were also found to be unimodally related to maximal decadal current speed, with diversity peaking at around 0.22 m/s (Figure 3h). A broadly similar form of relationship was observed between maximal decadal current speed and PR (*p* = .030), FR (*p* = .022), and taxon richness (*p* < .001); values of the three variables were found to peak at current speeds between ~0.18 m/s and 0.25 m/s, and then decline rapidly as maximal current speed increases further (Figure 3i). Maximal decadal current speeds were found to relate significantly with peracarid taxon assemblage structure (*p* < .001).

### 3.5 Physical disturbance and bottom trawling

**Figure A12:**
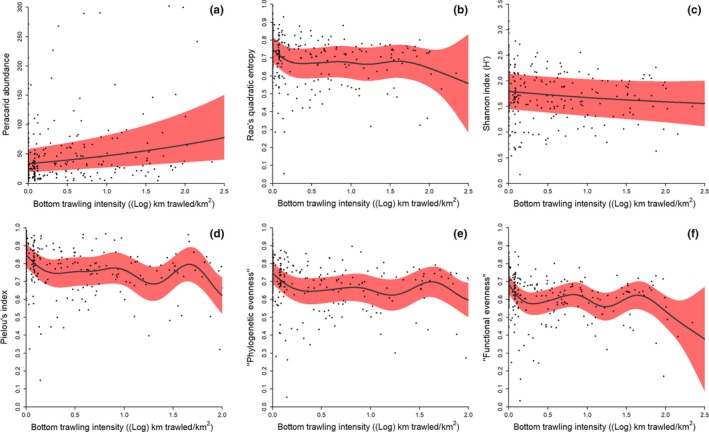
Partial relationships between bottom trawling intensity ((Log) km trawled/km^2^) at sampling locations (*n* = 312) in the Northwest Atlantic Ocean and different metrics of the biodiversity of peracarid assemblages sampled. Solid lines are smoothed lines of best fit as determined by multivariate generalized additive modeling. Colored bands are 95% confidence intervals. (a) Bottom trawling intensity/peracarid abundance; (b) bottom trawling intensity/Rao's quadratic entropy; (c) bottom trawling intensity/Shannon Index; (d) bottom trawling intensity/Pielou's Index; (e) bottom trawling intensity/phylogenetic evenness; (f) bottom trawling intensity/functional evenness

Bottom trawling intensity (Figure A12) was found to be positively related to peracarid abundance (*p* < .001; Figure 3j) but negatively related to FE (*p* < .001), PE (*p* < .001), Pielou's Index (*p* < .001), Rao's quadratic entropy (*p* = .004), and Shannon diversity (*p* = .044). These metrics generally exhibit a rapid initial decline with increasing trawling intensity, followed by undulation with an overall negative trend (Figure 3k). Peracarid taxon assemblage structure was also significantly related to trawling intensity (*p* < .001), with the cumacean genera *Diastylis* Say, 1818, *Diastyloides* G.O. Sars, 1900, and *Eudorella* Norman, 1867 relatively more abundant than other peracarid genera in samples collected from locations subjected to highest trawling intensity. The tanaidacean genera *Leptognathioides* Bird & Holdich, 1984, *Typhlotanais* Sars, 1882, and *Pseudosphyrapus* Gutu, 1980 were also relatively abundant in locations subject to high trawling intensity.

### 3.6 Temperature

**Figure A13:**
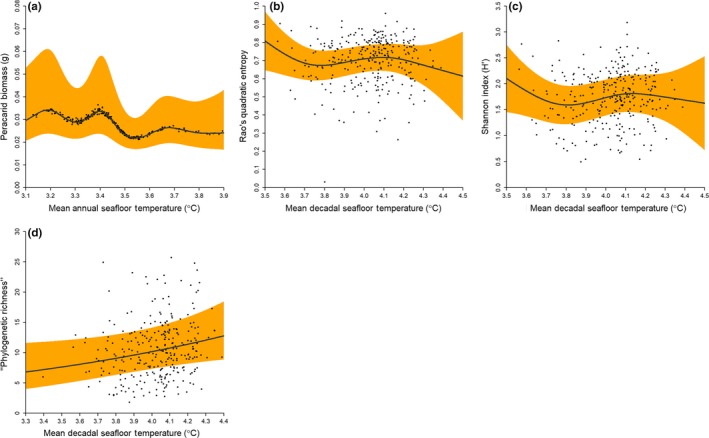
Partial relationships between mean annual/decadal seafloor temperature (°C) at sampling locations (*n* = 312) in the Northwest Atlantic Ocean and different metrics of the biodiversity of peracarid assemblages sampled. Solid lines are smoothed lines of best fit as determined by multivariate generalized additive modeling. Colored bands are 95% confidence intervals. (a) Mean annual temperature/peracarid biomass; (b) mean decadal temperature/Rao's quadratic entropy; (c) mean decadal temperature/Shannon Index; (d) mean decadal temperature/phylogenetic richness

Peracarid biomass was found to be related to average seafloor temperature for the year of sample collection in a complex, but overall negative manner (Figure A13a; *p* = .008). Rao's quadratic entropy (*p* = .072) and Shannon diversity (*p* = .062) showed weak evidence of an overall decline with increasing mean decadal seafloor temperature at sample locations (Figure A13). However, PR was found to increase with increasing mean decadal seafloor temperature (*p* = .038; Figure 3l). Average decadal seafloor temperature was significantly associated with peracarid taxon assemblage structure (*p* < .001).

### 3.7 Inter‐ and intra‐annual variability

**Figure A14:**
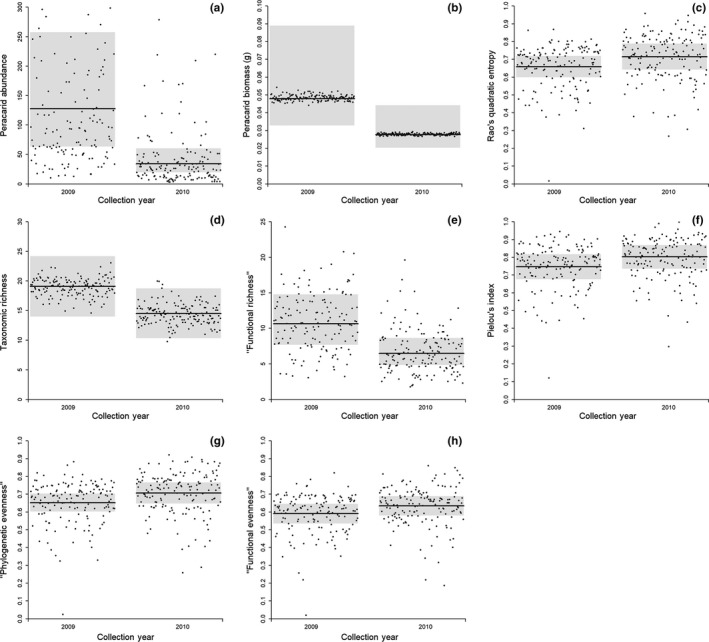
Partial relationships between collection year (2009/2010) and different metrics of the biodiversity of peracarid assemblages sampled from the Northwest Atlantic Ocean. Solid lines are mean values, and gray bands are 95% confidence intervals. (a) Collection year/peracarid abundance; (b) collection year/peracarid biomass; (c) collection year/Rao's quadratic entropy; (d) collection year/taxonomic richness; (e) collection year/functional richness; (f) collection year/Pielou's Index;(g) collection year/phylogenetic evenness; (h) collection year/functional evenness

Many of the biological metrics investigated varied by collection year (2009/2010; Figure A14). Peracarid abundance (*p* < .001), biomass (*p* = .003), FR (*p* = .011), and possibly taxon richness (*p* = .093) were reduced in 2010 relative to 2009, but PE (*p* = .001), FE (*p* = .031), Pielou's Index (*p* = .021), and Rao's quadratic entropy (*p* = .003) were all significantly elevated in 2010 relative to 2009.

**Figure A15:**
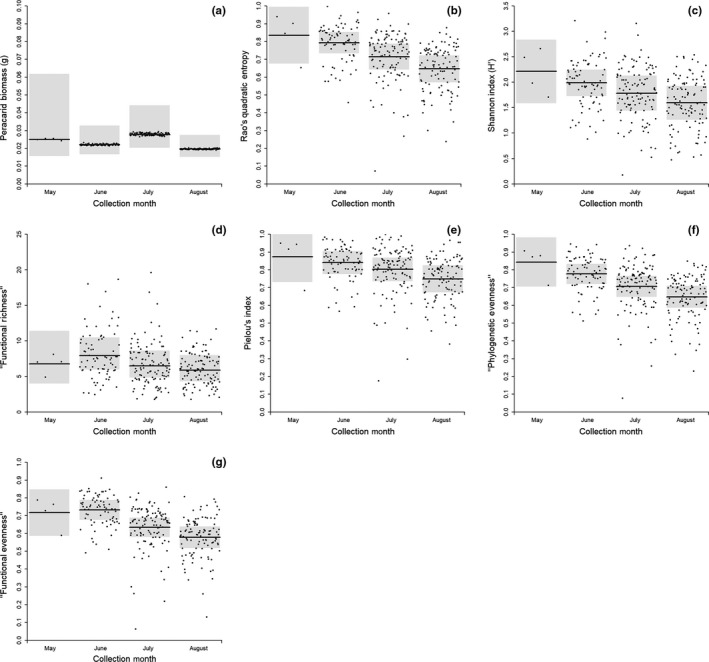
Partial relationships between collection month (May/June/July/August) and different metrics of the biodiversity of peracarid assemblages sampled from the Northwest Atlantic Ocean. Solid lines are mean values, and gray bands are 95% confidence intervals. (a) Collection month/peracarid biomass; (b) collection month/Rao's quadratic entropy; (c) collection month/Shannon Index; (d) collection month/functional richness; (e) collection month/Pielou's Index; (f) collection month/phylogenetic evenness; (g) collection month/functional evenness

Peracarid assemblage biodiversity metrics also varied with sample collection month (May/June/July/August; Figure A15), with FE (*p* < .001), PE (*p* < .001), Pielou's Index (*p* = .002), Rao's quadratic entropy (*p* < .001), Shannon diversity (*p* < .001), and possibly FR (*p* = .064) being elevated in May, June, and July, relative to August, and generally taking the form of a decline in values toward the summer. However, peracarid biomass reached a maximal value in July, relative to the other months that collections were made (*p* < .001).

**Figure 4:**
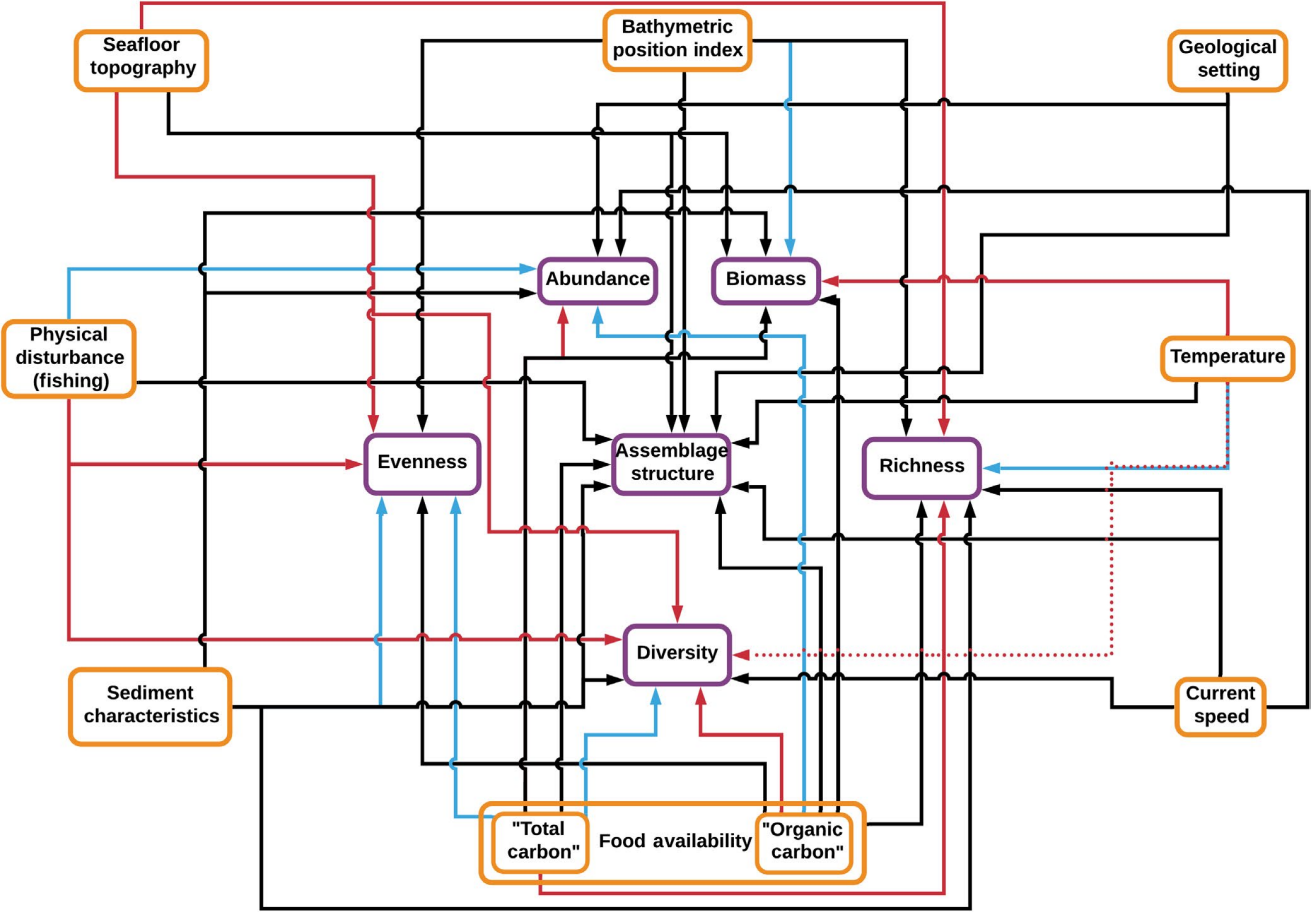
Summary of results based on analyses conducted in this study. Results are presented in a similar style to the conceptual model of Levin et al. (2001). Links between environmental parameters (orange boxes) and biological parameters (purple boxes) are specified. The color of arrow linking environmental and biological parameters represents form of relationship between the two: blue = positive, red = negative, black = complex (e.g., “U”‐shaped or uni/multimodal). Solid arrows represent significant relationships (*p* < .05). Dotted arrows represent marginal relationships (*p* < .1, >.05)

See Figure 4 and Tables 2 and 4 for a summary of the results of this investigation.

**Table 4:** Summary of results of statistical analyses conducted

	Abundance	Biomass	Rao's quadratic entropy	Shannon diversity	Taxon richness	PR	FR	Pielou's Index	PE	FE	Assemblage structure
Geological environment		ns	ns	ns	ns	ns	ns	ns	ns	ns	
Rugosity (1,875 × 1,875 m)	ns	ns			ns	ns		ns			
Roughness (225 × 225 m)	ns		ns	ns		ns	ns				ns
BPI (9,375 m radius)	ns		ns	ns			ns		ns		
Sediment PSD	ns		ns	ns	ns	ns	ns	ns	ns	ns	ns
Sediment sand content		ns									
Surface chlorophyll *a*					ns	ns	ns				
Surface POC			ns						ns	ns	
Seafloor POC	ns				ns	ns					
Sediment total carbon			ns							ns	
Sediment organic carbon	ns				ns						ns
Annual current speed		ns	ns	ns				ns	ns	ns	ns
Max decadal current speed		ns						ns	ns	ns	
Trawling intensity		ns			ns	ns	ns				
Annual temperature	ns		ns	ns	ns	ns	ns	ns	ns	ns	ns
Decadal temperature	ns	ns			ns		ns	ns	ns	ns	
Collection year				ns		ns					ns
Collection month	ns				ns	ns					ns

Note

Blue coloration denotes a positive relationship between dependent and independent variables. Red coloration denotes a negative relationship between dependent and independent variables. Black coloration denotes a complex (e.g., “U”‐shaped or uni/multimodal) relationship between dependent and independent variables. Dark color blocks represent significant relationships (*p* < .05). Light color blocks (light blue/light red/gray) represent marginal relationships (*p* < .1 > .05).

Abbreviations: BPI, Bathymetric Position Index; FE, Functional Evenness; FR, Functional Richness; ns, not significant; PE, Phylogenetic Evenness; POC, particulate organic carbon; PR, Phylogenetic Richness; PSD, particle‐size diversity.

## 4 DISCUSSION

### 4.1 Variability in seafloor topography

Prior observations concerning the importance of large‐scale habitat characteristics in determining assemblage biodiversity and structure (De Leo et al., 2010; Levin et al., 2010; Vetter & Dayton, 1998) are reiterated here by the finding of a significant relationship between geological environment and the density and structure of peracarid assemblages. However, our finding that elevated seafloor heterogeneity is related negatively with assemblage diversity (Table 4, Figure 4) is in contrast with the accepted influence of topographic variability on deep‐sea communities (Cordes et al., 2010; Levin et al., 2001; Vanreusel et al., 2010; Table 2). For example, elevated seafloor heterogeneity, as measured by high bathymetric variability at the hundreds to thousands of meter scale, was found to relate negatively with peracarid assemblage taxon richness, functional richness, phylogenetic evenness, functional evenness, and taxon diversity. Indeed, the only evidence that high topographic variability promotes increased faunal diversity is provided by the U‐shaped relationship between “roughness” and taxon evenness, with assemblage evenness declining to a minimum as seafloor roughness reaches moderate values, and then climbing again as those roughness values increase further.

It is possible that these unexpected relationships are linked to the life‐history traits of peracarid crustaceans. Peracarids brood their young and so have relatively limited dispersal capabilities compared to taxa with planktonic life stages. Therefore, topographic complexity over hundreds to thousands of meters may act as a dispersal barrier to peracarids. With reduced dispersal, individual meta‐populations will be smaller, and this could leave populations prone to local extinction because of the Allee effect (Lamont, Klinkhamer, & Witkowski, 1993; Stephens & Sutherland, 1999). Alternatively, the observed relationships could be explained by the fact that areas of elevated topographic heterogeneity are also characterized by increased disturbance frequency resulting from sediment instability. It is possible that the frequency and magnitude of these disturbances may limit assemblages to relatively early‐successional states characterized by reduced richness, evenness and diversity. That the taxonomic and functional metrics of diversity investigated were more closely related to topographic variables than the phylogenetic metrics were supports an ecological explanation, as opposed to an evolutionary explanation, of the influence of topographic variability on peracarid biodiversity.

It should be noted, however, that the measurement scale of topographic variability utilized here was limited by the resolution of the available bathymetric data (75 m cell size), which resulted in characterization of topographic variability at the hundreds to thousands of meter scale (Table 3). This spatial scale could be considered disjunct from the size of the samples analyzed (0.25 m^2^).

Our finding that peracarid biomass, richness (Figure 3a), and evenness vary depending on the elevation of a sampling point compared with its surrounds (“Bathymetric Position Index”; Table 2) builds upon a number of recent studies demonstrating increased taxon richness and faunal biomass on abyssal hills relative to plains or troughs for both meiofauna and megafauna (Durden et al., 2015; Morris et al., 2016; Simon‐Lledó et al., 2019; Stefanoudis et al., 2016). These studies link increased faunal biomass on hills, relative to plains, to elevated food availability as a result of topographically enhanced currents. However, we found no evidence for a positive relationship between current speed and peracarid biomass (Table 4, Figure 4), suggesting that another mechanism may be responsible for this observed pattern in the macrofaunal assemblages analyzed. That the taxonomic metrics of biodiversity were related more strongly to Bathymetric Position Index than the functional or phylogenetic metrics suggests a greater level of functional/phylogenetic similarity between ridges and valleys than taxonomic similarity.

### 4.2 Sediment characteristics

Our finding of the importance of sediment characteristics in determining the structure, abundance, evenness (Figure 3b), richness (Figure 3c), and diversity of benthic assemblages (Table 4, Figure 4) is in agreement with shallow‐water studies (Biernbaum, 1979; Cooper et al., 2011; Johnson, 1971; Table 2). Our results suggest that both moderately low and high proportions (~5%–15% and ~60%–80%, respectively) of sand‐sized particles are optimal in promoting maximal abundance and functional richness, phylogenetic richness, and taxon richness of benthic peracarid assemblages, with higher proportions of sand‐sized particles being more beneficial overall when taxon diversity and evenness, and functional and phylogenetic evenness are additionally considered.

These results are suggestive of two broad assemblage composition states that may be characterized by particle‐size preferences for feeding (Fenchel et al., 1975; Self & Jumars, 1988; Taghon, 1982) or perhaps burrow/tube construction. The first (slightly less diverse) assemblage is characterized by those taxa that preferentially utilize relatively small sediment particles (low sediment sand percent values), while the second (slightly more taxonomically and functionally diverse) assemblage is characterized by those taxa that preferentially use relatively large particles (high sediment sand percent). This hypothesis is supported by the finding of a significant relationship between sediment sand content and peracarid assemblage structure. That a strong minimum in the values of the biotic metrics analyzed does not occur at intermediate values of sediment sand content suggests that these two broad assemblages are not entirely distinct from one another.

That no relationship between particle‐size diversity and the richness or diversity of peracarid assemblages was found (Table 2) contradicts the results of Etter and Grassle (1992), Leduc, Rowden, Probert, et al. (2012), and Pape et al. (2013), but is in agreement with Lins, Silva, Neres, Esteves, and Vanreusel (2018). That particle‐size diversity was found to relate significantly with peracarid biomass, but not abundance, may suggest that changes in particle‐size diversity influence the body size of individuals.

Holistically, our results suggest that the peracarid assemblages investigated may be more greatly structured by average sediment particle size than they are by the specifics of particle‐size diversity.

### 4.3 Food availability

The variety of food availability metrics assessed provides a complex picture of the influence of food availability on deep‐sea peracarid assemblages (Table 4). However, these metrics can be divided into two general groupings based on the similarity of observed relationships with peracarid biodiversity. Grouping 1 (referred to henceforth as “total carbon” proxies) comprises surface particulate organic carbon (POC), seafloor POC, and sediment total carbon metrics, while grouping 2 (referred to henceforth as “organic carbon” proxies) comprises surface chlorophyll *a* and sediment organic carbon metrics. For example, we found increasing surface POC, seafloor POC, and total carbon to relate unimodally/negatively with peracarid biomass, and increasing surface POC and total carbon were both found to negatively affect peracarid abundance and richness metrics. Further, increasing surface chlorophyll *a* and organic carbon concentrations both exhibit a negative relationship with Rao's quadratic entropy, in contrast to the relationships obtained between surface POC, seafloor POC, and sediment total carbon metrics and Rao's quadratic entropy.

These two groupings demonstrate a notable similarity of results obtained from modeled surface food availability metrics, modeled seafloor food availability metrics, and actual measurements of carbon content from box core subsamples (Table 4). This was unexpected, since the modeled food availability metrics do not account for lateral movement of water between surface layers and the seabed, and disregard the potential importance of downslope transport of coastal primary production and riverine inputs, which, in some cases, may be of such importance that they represent the primary shaping factors of the underlying trophic regime (Johnson et al., 2007).

For “total carbon” proxies, we found high values to reduce the abundance, biomass, and richness (Figure 3e) of assemblages, while promoting increased evenness and possibly taxon diversity (Figure 4). These results are broadly the opposite of those expected, since deep‐sea environments are typically considered food‐limited (Corliss et al., 2009; Gooday et al., 1990; Johnson et al., 2007; Lambshead et al., 2002; Rex et al., 2006; Smith et al., 2008). Increased strength of predation may be a parsimonious explanation; elevated density‐dependent predation pressure in regions of higher food availability has been suggested to promote high levels of evenness and diversity, while suppressing overall deep‐sea faunal abundance and biomass (Dayton & Hessler, 1972; Rex, 1976, 1981).

“Organic carbon” proxies were not generally found to correlate as strongly as the “total carbon” proxies with the biodiversity metrics investigated. However, the relationships that were recovered contrast, to some extent, with those suggested by the “total carbon” metrics (Table 4, Figure 4). We found increases in the “organic carbon” metrics to correlate with increased peracarid abundance, while a U‐shaped relationship was recovered between “organic carbon” and biomass, a unimodal relationship with evenness (Figure 3f), and a negative relationship was identified with taxon diversity. Our use of phylogenetic and functional metrics of biodiversity uncovered relationships between “organic carbon” metrics and assemblage richness that would not have been identified where only taxon richness investigated (Table 4). This suggests an influence of “organic carbon” availability on the phylogenetic and functional structure of peracarid assemblages, even in the absence of a strong influence on taxon richness.

A unimodal relationship between deep‐sea diversity and productivity, consistent with the “Intermediate Productivity Theory” (Grime, 1973), has been reported by other studies (Cosson‐Sarradin et al., 1998; Jöst et al., 2019; Leduc, Rowden, Bowden, et al., 2012; Tittensor et al., 2011) and may help to explain our contrasting observations of positive, negative, and unimodal relationships between peracarid biodiversity and food availability (Table 2). If food availability in the study region is at a magnitude toward the peak of a theoretical unimodal curve, depending on the metric of food availability analyzed, our data may cover the incline, peak, or decline of this curve, giving observations of positive, unimodal, or negative productivity—diversity relationships.

Why the “total carbon” and “organic carbon” proxies provide differing conclusions remains unclear. Each proxy appears to indicate a different facet of food availability for the peracarid assemblages analyzed. Several studies have found a relatively low importance of labile detritus in deep benthic food webs (Iken, Bluhm, & Gradinger, 2005; van Oevelen, Soetaert, & Heip, 2012; Smith, Baldwin, Glatts, Kaufmann, & Fisher, 1998). For example, van Oevelen et al. (2012) reconstructed benthic carbon flows in the Porcupine Abyssal Plain using linear inverse modeling and concluded that labile detritus contributes <5% of the total carbon requirements for bacterial, meiofaunal, and macrofaunal components of the food web. It is possible that this reflects the rapid removal of fresh phytodetritus by mobile megafauna, such as holothurians (Ginger et al., 2001; Neto, Wolff, Billett, Mackenzie, & Thompson, 2006). It is conceivable that the “organic carbon” and “total carbon” groupings identified here reflect proxies for labile and more refractory carbon components, respectively. Details of the utilization of food by deep‐sea benthic macrofauna still require further investigation (Campanyà‐Llovet et al., 2017; van Oevelen et al., 2012).

### 4.4 Current speed

The relationships between seafloor current speed and the peracarid biodiversity metrics examined here largely agree with the unimodal form proposed by Levin et al. (2001). For instance, peracarid abundance (Figure 3g), taxon diversity (Figure 3h), and richness (Figure 3i) were found in general to describe a unimodal shape with increasing current speed, with values of these variables peaking in currents of around 0.15–0.25 m/s. Our results are in agreement with the accepted paradigm whereby faunas are depauperate at excessively low and high values of current speed (Levin et al., 2001; Table 2). At low current speeds, faunas may be depauperate because food and oxygen concentrations are physiologically limiting. At high current speeds, faunal abundance and diversity are also depressed, perhaps reflecting the winnowing of surface sediments, including removal of organic carbon, homogenization of sediment flow structures, and suppression of ecological succession (Levin et al., 2001). However, at moderate current speeds, macrofaunal communities may exhibit high levels of diversity and abundance, perhaps reflecting nonlimitation of food, assisted dispersal and a complexity of sediment flow structures (Levin et al., 2001).

### 4.5 Physical disturbance and bottom trawling

Our finding that trawling intensity alters assemblage structure and is positively related to peracarid abundance (Figure 3j), but negatively related to evenness (Figure 3k) and taxon diversity, is generally consistent with the few studies that have investigated or reviewed trawling impacts on deep‐sea macrofauna and meiofauna (Clark et al., 2016; Clark & Rowden, 2009; Koslow & Gowlet‐Holmes, 1998; Koslow et al., 2001; Pusceddu et al., 2014; Román et al., 2016; Table 2). Common to the form of response of evenness and diversity metrics to increasing bottom trawling intensity was a rapid initial decline followed by undulation with an overall negative gradient (Figure 3k). It is possible that this undulation reflects turnover in assemblage structure with increasing trawling intensity.

Our results may be explained by invoking the intermediate disturbance hypothesis (Connell, 1978); the repeated passage of a trawl represents a high magnitude disturbance and results in a reduction in faunal diversity. Since no relationship between bottom trawling intensity and any measure of assemblage richness was uncovered, the observed declines in evenness and diversity likely reflect relative increases in the abundance of particular taxa that are less sensitive to disturbance. This is supported by a slightly elevated peracarid abundance and altered assemblage taxon structure with increasing trawling intensity and suggests that trawling has a filtering effect on the deep‐sea benthic community (Ashford et al., 2018).

However, the impacts of bottom trawling on the deep seafloor extend beyond physical disturbance. Increased bottom trawling frequency, and associated sediment resuspension, has been shown to reduce the bioavailability of carbon (Pusceddu et al., 2014, 2005), and this can negatively impact benthic meiofaunal diversity (Pusceddu et al., 2014). There may be some signal of this in the present dataset, with reductions in evenness and taxon diversity, but elevated faunal abundance mirroring the assemblage responses recovered for reductions in the “total carbon” proxies discussed above.

Fishing, similar to variation in food availability, can influence predator/prey relationships (Frank, Petrie, Fisher, & Leggett, 2011; Perez‐Rodriguez, Koen‐Alonso, & Saborido‐Rey, 2012; Shepherd & Myers, 2005) because of its direct removal of large mobile predators. Peracarid crustaceans represent an important dietary component of many fish species, including ones of commercial value (Modica, Cartes, & Carrassón, 2014). In the sampling area covered by this study, peracarids are a prey item of small (<20 cm) Greenland Halibut (*Reinhardtius hippoglossoides* [Walbaum, 1792]; Bowering & Lilly, 1992). Considering this, in contrast to the suggested role of predation in influencing levels of evenness and diversity with variation in food availability, the observed changes in peracarid assemblages with increasing trawling intensity (increased abundance, reduced evenness, and diversity) could represent a signature of the release of particular prey taxa. Our ordination results suggest the identity of these groups to be the cumacean genera *Diastylis*, *Diastyloides*, and *Eudorella*, and the tanaidacean genera *Leptognathioides*, *Typhlotanais*, and *Pseudosphyrapus*. However, as this is a correlational study, and correlation does not necessarily imply causation, the potential impacts of fishing reported here may in fact be attributable to another variable that is closely correlated with fishing effort but has not been incorporated into the models analyzed in this study. Experimental work is required to clarify the causative mechanisms between increasing trawling intensity and changes to peracarid biodiversity metrics.

### 4.6 Temperature

Our finding that high seafloor temperature values have a negative impact on the biomass and possibly taxon diversity of peracarid assemblages is in agreement with the recognition of temperature as a fundamental controlling environmental factor in marine environments (Danovaro et al., 2004; Hunt, Cronin, & Roy, 2005; Jöst et al., 2019; Perry, Low, Ellis, & Reynolds, 2005; Poloczanska et al., 2013; Tittensor et al., 2010; Yasuhara & Danovaro, 2016), and with the potentially high sensitivity of deep‐sea organisms to temperature change (Howes et al., 2015; McCauley et al., 2015; Tewksbury et al., 2008; Yasuhara et al., 2009). Our finding of a negative relationship between temperature and peracarid biomass may reflect the narrow thermal niche of many deep‐sea species (Carney, 2005; Yasuhara & Danovaro, 2016). This is supported by our finding of a significant relationship between temperature and assemblage structure (Figure 4). An alternative explanation is that this relationship reflects increased individual metabolic rates under higher temperatures (Brown et al., 2004; Jones et al., 2014). An implication of this result is that an increase in ocean temperature (particularly at continental slope depths [Balmaseda, Trenberth, & Kallen, 2013; Howes et al., 2015; Llovel, Willis, Landerer, & Fukumori, 2014; Ramirez‐Llodra et al., 2011]) may result in depressed ecosystem functioning. For example, a reduction in the biomass of organic carbon‐rich peracarids may suppress benthic carbon storage, this being of significance because of the importance of continental slopes for carbon storage globally (Levin & Dayton, 2009; Levin & Sibuet, 2012; Muller‐Karger et al., 2005; Rogers, 2015; Sweetman et al., 2017).

In agreement with the theory that temperature may only significantly influence seafloor diversity when at relatively high and low levels (Yasuhara & Danovaro, 2016), no strong relationship between temperature and any of the diversity or evenness metrics investigated was found. It is possible that this reflects the relatively small temperature range of approximately one degree Celsius investigated here. This temperature range may not be large enough for many temperature—biodiversity patterns to be clearly expressed. Alternatively, it has been argued that temperature may not exert a strong influence over deep‐sea biodiversity patterns on ecological timescales (McClain et al., 2012; Woolley et al., 2016). Supporting this, temperature was found to relate positively with phylogenetic richness (Figure 3l), but not taxon or functional richness. This may suggest that temperature has a stronger influence over evolutionary processes, such as speciation and extinction, than it does over ecological processes controlling coexistence.

### 4.7 Inter‐ and intra‐annual variability

That the peracarid assemblages were found to vary both intra‐ and interannually builds on the findings of previous studies that record seasonality in deep‐sea fauna (Corliss et al., 2009; Gooday, 1988; Graf, 1989; Smith & Baldwin, 1984). Reduced peracarid abundance, biomass, functional richness, and possibly taxon richness, but elevated taxon evenness, functional evenness, phylogenetic evenness, and taxon diversity in 2010 relative to 2009 may be related to the extreme Northern Hemisphere winter that occurred between the two years as a result of a negative North Atlantic Oscillation (Fereday, Maidens, Arribas, Scaife, & Knight, 2012; Seager, Kushnir, Nakamura, Ting, & Naik, 2010). According to the datasets analyzed in this study, while temperature and salinity do not differ greatly between 2009 and 2010 at the sampled locations (2009 mean temperature = 3.40°C, standard error = ±0.01; 2010 mean temperature = 3.53°C, standard error = ±0.01; 2009 mean salinity = 34.69‰, standard error = ±0.01; 2010 mean salinity = 34.72‰, standard error = ±0.01), surface food availability metrics differ more dramatically (2009 mean surface POC concentration = 133.2 mg/m^3^, standard error = ±0.75; 2010 mean surface POC concentration = 156.5 mg/m^3^, standard error = ±0.76). Other environmental factors may have differed between the sampling years, but this difference in food availability may explain in part the faunal differences observed.

Observed trends of declining evenness and taxon diversity from May to August may reflect a signature of the seasonal pulsing of labile organic matter to depth that occurs in temperate regions (Billett, Lampitt, Rice, & Mantoura, 1983; Ittekkot, Deuser, & Degens, 1984; Rice et al., 1986). This phenomenon has been called upon in past studies to explain similar patterns (Corliss et al., 2009; Gooday, 1988). The observed changes in the above biological variables are suggestive of an increase in the relative dominance of particular functional and phylogenetic groups as high‐quality food becomes more plentiful between spring and summer (Campanyà‐Llovet et al., 2017; Ginger et al., 2001; Neto et al., 2006). In total, our results suggest that deep‐sea benthic communities are not greatly buffered from surface and atmospheric processes.

### 4.8 Functional convergence

The functional and phylogenetic metrics examined here did not always provide similar results (Table 4). For example, Bathymetric Position Index was found to relate significantly with functional evenness but not phylogenetic evenness, and seafloor temperature was significantly related to the phylogenetic richness, but not the functional richness of the macrofaunal assemblages analyzed. These observations further suggest that, as highlighted by Ashford et al. (2018), for peracarid crustaceans, phylogeny is not a perfect proxy for functional similarity, with functional convergences sometimes occurring between distantly related taxa.

### 4.9 Comparison of results with conceptual model of Levin et al. (2001), and study limitations

When considering only taxon richness, we find our results to both corroborate and challenge the hypotheses put forward by Levin et al. (2001), depending on the environmental variable in question. For example, Levin et al. (2001) propose a unimodal relationship between physical disturbance and species richness, while we found no significant relationship between the two variables; Levin et al. (2001) propose a positive relationship between seafloor heterogeneity and species richness, and a unimodal relationship between food availability and species richness, whereas we found a negative relationship between taxon richness and both topographic heterogeneity and food availability. However, our results support the unimodal relationship between taxon richness and current speed proposed by Levin et al. (2001).

These differences may in part be explained by the differential scope of the two studies. While Levin et al. (2001) aimed to summarize predicted relationships between the environment and species richness across a selection of taxonomic groups and ocean basins, our study aims to statistically investigate these relationships, and is more restricted in its taxonomic and spatial scope, considering only peracarid crustaceans and data only from the Northwest Atlantic Ocean at bathyal depths. Peracarid crustaceans are very abundant, highly diverse, and ecologically important in deep‐sea sediments (Larsen, Tuya, & Froufe, 2014; Poore, 2005; Spears, Debry, Abele, & Chodyla, 2005), yet they constitute only a limited component of deep‐seafloor communities. By investigating only peracarids, we may have failed to fully recognize the role biological interactions with other components of deep‐seafloor communities play on patterns of deep‐ocean biodiversity. Similarly, while the geographical extent of our sampling is very large for a study of this type, our data characterize only a single region of the world's oceans. The variability in environmental parameters investigated therefore does not encompass the full degree of variety of deep‐sea environments that occur across the globe. Contrasting conclusions may be made if data from alternative locations and macrofaunal taxonomic subsets were to be analyzed.

It is important to note that our analyses were not undertaken at the species level. The unavailability of phylogenetic and functional information for deep‐sea peracarid crustaceans at the species level forced us to work at higher taxonomic levels in order to be able to include phylogenetic and functional metrics of biodiversity. Further, the scale of the study coupled with the large number of undescribed species in the deep ocean (e.g., see Poore et al., 2015), and the scarcity of suitable keys and taxonomic literature made identification prohibitively time‐intensive. Our use of higher taxonomic levels contrasts with much of the theory presented, which generally assumes species to be the taxonomic unit in question. It is not clear how appropriately this theory can be applied to higher taxonomic levels, such as genera, and this may partially explain why some of our results contrast with accepted paradigms. However, numerous studies provide confidence that quantifying diversity at higher taxonomic levels, particularly at the genus level, can provide an effective proxy for species diversity (Balmford, Green, & Murray, 1996; Jablonski & Finarelli, 2009; La Ferla, Lovett, Ockwell, & Taplin, 2002; Roy, Jablonski, & Valentine, 1996; Villaseñor, Ibarra‐Manríquez, Meave, & Ortíz, 2005).

Our analyses included environmental variables derived from different sources at different spatial scales. For example, percent sand content was measured directly from box core subsamples, while average annual current speed was calculated from modeled data at a spatial resolution of 578 × 578 m (Ashford et al., 2018). It is possible that, were all environmental variables measured across the same spatial scale, different results would have been obtained, as studies have shown that the influence of environmental parameters on biodiversity may depend on the scale of measurement (Økland, Bakke, Hågvar, & Kvamme, 1996; Schiegg, 2000; Tews et al., 2004). That we find a significant influence of variables measured at a variety of spatial scales on peracarid biodiversity is an interesting result in itself, however, because it suggests that peracarid biodiversity is influenced by a combination of environmental factors acting over multiple spatial scales. For example, our results suggest that the observed taxon richness of peracarids in the study area is influenced by a combination of BPI (characterized at the kilometer scale), seafloor roughness, surface POC concentration, and average and maximum current speeds (characterized at the hundreds of meter scale), and sediment sand and total carbon percent content (characterized at the centimeter scale; Table 4). However, if we compare the influence on peracarid biodiversity of variables representing similar environmental facets but measured at different spatial scales, we find a similarity of results, which suggests that any influence of varying measurement spatial scale on our results is unlikely to be large. For example, the influences of surface POC concentration and sediment percent total carbon on peracarid biodiversity were found to be nearly identical (Table 4) despite their contrasting spatial measurement scales.

### 4.10 An expanded concept of biodiversity

In this study, we aimed to investigate an expanded concept of “biodiversity,” relative to typical deep‐sea studies, additionally quantifying functional and phylogenetic facets of diversity alongside more traditional taxonomic measures. Our broad concept of biodiversity, the differences in results between the different diversity metrics analyzed (Table 4), and the similarities and differences between the conceptual model of Levin et al. (2001) and our results emphasize the importance of quantifying a variety of biodiversity metrics when investigating benthic macrofaunal assemblages, and their relationships with environmental variables (Pape et al., 2013). For instance, the impacts of direct and indirect anthropogenic disturbance on deep‐ocean communities, such as climate change and deep‐seabed mining, may only be fully realized when an array of biodiversity metrics are investigated. We find trawling intensity to relate negatively with taxonomic, functional, and phylogenetic evenness, and positively with faunal abundance, but not to relate significantly with assemblage richness, for example. Further, we find evidence for a significant positive relationship between seafloor temperature and phylogenetic richness that would not have been identified if only taxon richness were investigated.

We therefore call for efforts to be made to investigate an expanded array of metrics in future studies that aim to characterize deep‐ocean biodiversity, and we particularly emphasize the value of quantifying functional and phylogenetic measures of diversity, in addition to the more traditional taxonomic metrics.

## 5 CONCLUDING REMARKS

Despite the fact that deep‐sea benthic communities cover more than 90% of seafloor area, host countless species and contribute significantly to essential ecosystem services of global relevance, knowledge of the ecology of deep‐sea soft‐sediment communities remains limited. Here, we aimed to progress understanding of the intricate relationships between deep‐sea benthic macrofaunal biodiversity and the deep‐ocean environment by the consideration of a large array of peracarid biodiversity metrics and a variety of environmental variables. We found food availability, physical disturbance (trawling intensity), and current speed to be the three variable sets that were most consistently related to metrics of peracarid biodiversity (Table 4). Our results challenge a selection of prevailing hypotheses of deep‐seafloor biodiversity. For example, seafloor topographic heterogeneity at the scale of hundreds to thousands of meters was found to be associated with reduced peracarid biodiversity, while sediment particle‐size diversity was found to be related less strongly to assemblage attributes than broadscale sediment characteristics such as sand content. However, our results also lend support to a number of longstanding paradigms, such as that deep‐sea assemblages may vary intra‐ and interannually, and how assemblages respond to changes in seafloor current speed.

The results of our investigation provide some evidence that climate change may significantly influence deep‐sea community biomass and that bottom trawling negatively affects the evenness and diversity of deep‐sea soft‐sediment peracarid assemblages. Our results emphasize that deep‐sea benthic ecosystems are not highly buffered from surface and atmospheric processes, and indeed, it appears likely that predicted changes to surface and atmospheric environments over the coming decades will propagate through the water column and alter the characteristics of deep‐sea benthic communities, this having concomitant effects on the provision of ecosystem services.

Finally, our study demonstrates value in investigating a broad array of biodiversity metrics, and we therefore call for researchers to consider an expanded definition of biodiversity in future investigations of deep‐ocean life.

## CONFLICT OF INTEREST

Author C.R.S.B.F. is employed by company Seascape Consultants Ltd. All other authors declare no competing interests.

## AUTHOR CONTRIBUTIONS

This study was conceived by O.S.A., A.D.R., A.J.K., and C.R.S.B.F., A.D.R., A.J.K., and C.R.S.B.F. provided biological specimens, access to environmental data, and secured funding. O.S.A. and T.H. identified the biological specimens. O.S.A. and C.R.S.B.F. processed the environmental data. O.S.A. undertook all analyses and wrote the manuscript, which was contributed to and edited by A.J.K., C.R.S.B.F., T.H., and A.D.R.

## Data Availability

All data utilized by this study are publicly available in the Supporting Information (Tables S1 and S2, Data S1 and S2) section of the manuscript and via “figshare” (https://doi.org/10.6084/m9.figshare.10120151).
