# Supplementary material for: Investigating the environmental drivers of deep‐seafloor biodiversity: A case study of peracarid crustacean assemblages in the Northwest Atlantic Ocean
Source: Ecol Evol. 2019 Nov 27;9(24):14167–204. doi: 10.1002/ece3.5852 (PMC6953587; doi:10.1002/ece3.5852)
Supplement: Supplementary file 1 [file ECE3-9-14167-s001.docx]

**‘****Investigating the environmental drivers of deep-seafloor biodiversity: a case study of peracarid crustacean assemblages in the Northwest Atlantic Ocean’**

**Supporting Information**

**Oliver S. Ashford ^1,2,†*^, Andrew J. Kenny ^2^, Christopher R. S. Barrio Froján ^3^, Tammy Horton ^4^ & Alex D. Rogers ^1^**

^1^ Department of Zoology, University of Oxford, Oxford, OX1 3PS, UK.

^2^ Centre for the Environment, Fisheries and Aquaculture Science (Cefas), Pakefield Road, Lowestoft, NR33 0HT, UK.

^3^ Seascape Consultants Ltd, Romsey, SO51 0PE, UK.

^4^ National Oceanography Centre, University of Southampton Waterfront Campus, European Way, Southampton, SO14 3ZH, UK.

^†^ Present address: Sverdrup Hall, 8615 Kennel Way, Scripps Institution of Oceanography, La Jolla, California, 92037, USA.

* Corresponding author: oashford@ucsd.edu. Mobile telephone: (+1) 858-405-4298.

**Table S1:** Correlation matrix of all continuous environmental variables analysed in this study following Variance Inflation Factor analysis (Pearson’s correlation coefficient) (see Table 3). Colours represent correlation strength; dark green=0.0 to <0.25/−0.25), light green=0.25 to <0.50/−0.25 to −0.50), orange=0.50 to <0.75/−0.50 to −0.75).

|  | **Annual temperature** |  |  |  |  |  |  |  |  |  |  |  |  |  |  |
| --- | --- | --- | --- | --- | --- | --- | --- | --- | --- | --- | --- | --- | --- | --- | --- |
| **Annual temperature** | 1.00 | **Decadal temperature** |  |  |  |  |  |  |  |  |  |  |  |  |  |
| **Decadal temperature** | 0.16 | 1.00 | **Annual current speed** |  |  |  |  |  |  |  |  |  |  |  |  |
| **Annual current speed** | -0.05 | 0.01 | 1.00 | **Decadal current speed** |  |  |  |  |  |  |  |  |  |  |  |
| **Decadal current speed** | -0.36 | -0.53 | 0.01 | 1.00 | **Surface chlorophyll *a*** |  |  |  |  |  |  |  |  |  |  |
| **Surface Chlorophyll *a*** | 0.30 | 0.01 | -0.09 | -0.23 | 1.00 | **Surface POC** |  |  |  |  |  |  |  |  |  |
| **Surface POC** | 0.19 | 0.07 | 0.30 | -0.20 | 0.62 | 1.00 | **Seafloor POC** |  |  |  |  |  |  |  |  |
| **Seafloor POC** | 0.07 | 0.57 | -0.13 | -0.35 | 0.01 | 0.03 | 1.00 | **Percent total carbon** |  |  |  |  |  |  |  |
| **Percent total carbon** | 0.15 | -0.52 | -0.19 | 0.07 | 0.00 | -0.05 | -0.28 | 1.00 | **Percent organic carbon** |  |  |  |  |  |  |
| **Percent organic carbon** | -0.05 | -0.09 | 0.39 | 0.03 | 0.06 | 0.18 | -0.34 | -0.19 | 1.00 | **Percent sand** |  |  |  |  |  |
| **Percent sand** | -0.16 | 0.20 | 0.27 | 0.00 | -0.24 | 0.06 | 0.42 | -0.14 | -0.28 | 1.00 | **Sediment PSD** |  |  |  |  |
| **Sediment PSD** | 0.09 | -0.07 | 0.24 | -0.07 | -0.04 | 0.04 | -0.22 | 0.01 | 0.36 | -0.09 | 1.00 | **Log(trawling intensity)** |  |  |  |
| **Log(trawling intensity)** | -0.01 | 0.35 | -0.25 | -0.10 | 0.11 | 0.00 | 0.30 | -0.29 | -0.24 | 0.02 | -0.36 | 1.00 | **Bathymetric position index** |  |  |
| **Bathymetric position index** | -0.09 | 0.03 | -0.01 | 0.07 | -0.03 | 0.00 | 0.46 | -0.11 | -0.30 | 0.36 | -0.24 | 0.09 | 1.00 | **Seafloor rugosity** |  |
| **Seafloor rugosity** | -0.13 | 0.06 | 0.20 | -0.04 | 0.02 | 0.28 | -0.14 | -0.22 | 0.27 | 0.15 | 0.07 | -0.07 | -0.31 | 1.00 | **Seafloor roughness** |
| **Seafloor roughness** | -0.28 | 0.01 | 0.36 | 0.11 | -0.07 | 0.18 | -0.04 | -0.24 | 0.24 | 0.39 | 0.04 | -0.09 | 0.03 | 0.46 | 1.00 |

**Table S2:** Details of independent variables (see Table 3), and error distribution and link functions (where appropriate) used for each optimised Generalised Additive Model and Constrained Analysis of Principal coordinates model constructed.

| Peracarid biodiversity metric | Independent variables in optimal model | Error distribution and link function | Comments |
| --- | --- | --- | --- |
| Abundance | Annual current speed; decadal current speed; surface chlorophyll *a*; surface POC; percent total carbon; percent sand content; geological environment; trawling intensity; collection year | Negative binomial, log | Negative binomial model structure used as original Poisson model exhibited unacceptable levels of overdispersion (theta >30) |
| Biomass | Annual temperature; decadal temperature; surface chlorophyll *a*; surface POC; seafloor POC; percent total carbon; percent organic carbon; sediment particle size diversity; bathymetric position index; seafloor roughness; collection year; collection month | Gamma, inverse |  |
| Rao Quadratic Entropy | Decadal temperature; decadal current speed; surface chlorophyll *a*; seafloor POC; percent organic carbon; percent sand content; trawling intensity; seafloor rugosity; seafloor roughness; collection year; collection month; crew identity | Gaussian, identity |  |
| Shannon Diversity | Decadal temperature; annual current speed; decadal current speed; surface chlorophyll *a*; surface POC; seafloor POC; percent total carbon; percent organic carbon; percent sand content; trawling intensity; seafloor rugosity; collection month; crew identity | Gaussian, identity |  |
| Taxon Richness | Annual current speed; decadal current speed; surface POC; percent total carbon; percent sand content; bathymetric position index; seafloor roughness; collection year | Quasi-Poisson, identity | Quasi-Poisson model structure used as original Poisson model exhibited an overdispersion parameter value of 2.84 |
| Phylogenetic Richness (PR) | Decadal temperature, annual current speed; decadal current speed; surface POC; percent total carbon; percent organic carbon; percent sand content; bathymetric position index; collection year | Gamma, log |  |
| Functional Richness (FR) | Annual current speed; decadal current speed; surface POC; seafloor POC; percent total carbon; percent organic carbon; percent sand content; seafloor rugosity; collection year; collection month | Gamma, log |  |
| Pielou’s Index | Surface chlorophyll *a*; surface POC; seafloor POC; percent total carbon; percent organic carbon; percent sand content; sediment particle size diversity; trawling intensity; bathymetric position index; seafloor rugosity; seafloor roughness; collection year; collection month; crew identity | Gaussian, identity |  |
| Phylogenetic Evenness (PE) | Surface chlorophyll *a*; seafloor POC; percent total carbon; percent organic carbon; percent sand content; trawling intensity; seafloor rugosity; seafloor roughness; collection year; collection month; crew identity | Gaussian, identity |  |
| Functional Evenness (FE) | Surface chlorophyll *a*; surface POC; seafloor POC; percent total carbon; percent organic carbon; percent sand content; trawling intensity; bathymetric position index; seafloor rugosity; seafloor roughness; collection year; collection month; crew identity | Gaussian, identity |  |
| Assemblage Structure | Decadal current speed; decadal temperature; surface chlorophyll *a*; surface POC; seafloor POC; percent total carbon; percent sand content; trawling intensity; geological environment; bathymetric position index; seafloor rugosity; collection year; collection month; crew identity | NA |  |
